# cGAS–STING pathway modulation: A new hope for neural regeneration

**DOI:** 10.4103/NRR.NRR-D-24-01516

**Published:** 2025-08-13

**Authors:** Jinghan Zhang, Mouyuan Sun, Yaxian Luo, Mikko Petteri Räisänen, Lianjie Peng, Luying Qin, Mengfei Yu, Haifei Shi

**Affiliations:** 1 Department of Orthopedics, the First Affiliated Hospital of Zhejiang University School of Medicine, Hangzhou, Zhejiang Province, China; 2 Stomatology Hospital, School of Stomatology, Zhejiang University School of Medicine, Zhejiang Provincial Clinical Research Center for Oral Diseases, Key Laboratory of Oral Biomedical Research of Zhejiang Province, Cancer Center of Zhejiang University, Engineering Research Center of Oral Biomaterials and Devices of Zhejiang Province, Hangzhou, Zhejiang Province, China; 3 Department of Orthopedics, Traumatology, and Hand Surgery, Kuopio University Hospital, Kuopio, Finland

**Keywords:** biomaterials, cGAS–STING, nerve injury, neural regeneration, neurodegeneration, neuroinflammation, precision medicine, tissue engineering

## Abstract

In recent decades, the limitations of therapeutic interventions have elevated neurological disorders and injuries to a prominent position in academic research. Existing neurotherapeutic methodologies have demonstrated insufficient efficacy in fostering neural regeneration. The current integration of precision medicine technologies and innovative tissue engineering methods holds significant promise for attaining neural regeneration. The cGAS–STING pathway, a pivotal component of the innate immune system, plays a crucial role in the pathological processes of various neurological diseases and injuries. In neuroinflammatory diseases and neural injuries, aberrant activation of the cGAS–STING pathway amplifies neuroinflammation, type I interferon responses, and cell death. Inhibition of cGAS–STING-related genes holds promise for promoting neural regeneration following disease recovery and defect regeneration. In this review, the foundational pathophysiological mechanisms underlying cGAS–STING-related gene regulation in neurological disorders and injuries are elucidated with a special emphasis on its implications in nerve-related cells. In this review, we highlight the advances in tissue engineering technologies that integrate cGAS–STING pathway modulators, highlighting their potential therapeutic efficacy in modulating neural regeneration. Nevertheless, the role of the cGAS–STING pathway in neural regeneration remains relatively limited. Bibliometric analysis demonstrates a significant correlation of cGAS–STING pathway activation with various neuropathological processes. Studies have progressively focused on the critical role of this pathway in neurological diseases and injuries. As it stands, the effectiveness of tissue engineering technologies involving cGAS–STING-related gene modulators in achieving neural regeneration remains unfulfilled in its potential. Future research must apply advanced omics technologies to further delineate the exact functions of the cGAS–STING pathway in neural regeneration. Integration of these results with precision medicine approaches will be necessary for creating tissue engineering biomaterials with capabilities for precise delivery and targeted controlled release of cGAS–STING-related genes in neural regeneration-related cells, towards functional recovery from neurological injury and diseases.

## Introduction

The cGAS–STING pathway is a crucial regulator of innate immune signaling and is heavily involved in many neurological disorders and injuries (Xu et al., 2024b; Huang et al., 2025a). cGAS, the primary cytosolic sensor for DNA, is able to detect both exogenous pathogen DNA and endogenous DNA that is liberated from cellular damage. This detection triggers STING, which subsequently activates type I interferon (IFN-I) signaling and the production of pro-inflammatory cytokines (Yang et al., 2024c), which act to neutralize pathogens and coordinate immune responses. The cGAS–STING pathway has been identified as an important mechanism for the pathogenesis of neurological diseases and injuries (Wang et al., 2024e).

The nervous system is a precisely regulated and complex ecosystem where precise regulation of immune responses is crucial to maximize neuronal recovery of function and permit neural regeneration (Enamorado et al., 2023). Hyperactivation of the cGAS–STING pathway leads to increased neuroinflammation and neurodegeneration, which promotes disease progression and impedes neural regeneration (Gulen et al., 2023; Liu et al., 2025c). Recent reports have demonstrated the presence and functional significance of cGAS–STING signaling in various cell types of the nervous system, including resident immune cells, neurons, and glial cells (Verma et al., 2024). Currently, therapeutic reagents against the cGAS–STING pathway have been developed for the therapy of neural injuries and neuroinflammatory diseases to alleviate neuroinflammation and promote the restoration of neurological function (Huang et al., 2023; Lu et al., 2025b). However, there is no systematic review that synthesizes the cGAS–STING pathway mechanisms and its applicable targeted therapeutic material in neurological diseases and injuries.

This review summarizes the current research status and treatment methods including the cGAS–STING pathway in neurological diseases and injuries, indicating their limitations and suggesting directions for future research on mechanisms and the establishment of treatment methods. Through bibliometric analysis, we have extracted the research hotspots and key events in this field, highlighting the most important role played by genes associated with cGAS–STING in neurological injuries and diseases. It also hopes to give directions for future studies of the mechanisms involved in and therapeutic strategy design with cGAS–STING in these disorders. By conducting the clarification of the role of cGAS–STING in neurological disorders and injury, we hope to encourage future studies on related mechanisms and propose new directions of research on the application of tissue engineering technologies in the treatment of neurological diseases and injuries.

## Overview of the cGAS–STING Pathway

cGAS–STING pathway, a quintessential component of innate immunity, perceives cytosolic DNA to induce the activation of inflammatory cytokines and IFN-Is to promote immunity. It is also crucial for intercellular communication and cellular perturbation sensing, contributing significantly to various pathophysiological processes.

### The components of the cGAS–STING pathway

**Figure 1:**
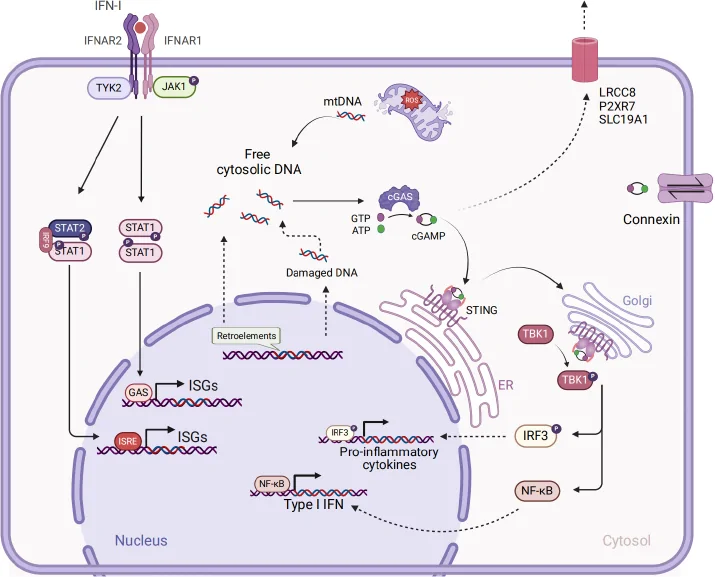
Overview of the cGAS–STING pathway. Cytoplasmic DNA from internal and external sources is detected by cGAS, forming a 2:2 complex. cGAS then synthesizes cGAMP using ATP and GTP. cGAMP binds to STING on the ER, triggering its migration to the Golgi. This recruits and activates kinases TBK1 and IKK, which activate IRF3 and NF-κB. This reaction cascade leads to IFN-I and inflammatory factor expression, bolstering immune responses. Subsequently, IFN-I can activate the expression of ISGs through the JAK-STAT pathway. Additionally, cGAMP can facilitate intercellular signaling via connexins and transmembrane proteins, including LRCC8, P2XR7, and SLC19A1. Created with BioRender.com (https://BioRender.com/sy0zfr7). ATP: Adenosine triphosphate; cGAMP: cyclic GMP-AMP; cGAS: cyclic GMP-AMP synthase; ER: endoplasmic reticulum; GTP: guanosine triphosphate; IFN: interferon; IRF3: IFN regulatory factor 3; JAK: Janus kinase-signal transducer; NF-κB: nuclear factor kappa B; STAT: signal transducer and activator of transcription; STING: stimulator of interferon genes; TBK1: TANK-binding kinase 1.

The cGAS–STING pathway provides a critical connection between DNA detection and the innate immune system (Kirchhoff et al., 2025). This pathway involves genes such as cGAS, STING, nuclear factor kappa B (NF-κB), IFNs, Janus Kinases (JAK), and signal transducers and activators of transcription (STAT) (**Figure 1**), which play pivotal roles in neuroinflammation, neurodegeneration, and neuroimmune functions (Hou et al., 2021; Lu et al., 2025b). Above all, the cGAS–STING pathway acts on immune cells and activates inflammatory cytokine secretion that aggravates neuroinflammation (Shi et al., 2023; Wang et al., 2024d; Zhou et al., 2025b; Liu et al., 2025c). In addition to influencing immune cells, activation of the cGAS–STING pathway is also observed in non-immune cells such as neurons and glial cells, where it regulates their cellular processes (Jiang et al., 2023a).

cGAS functions as a DNA sensor through the recognition of double-stranded DNA (dsDNA) in the cytoplasm and the catalysis of production of the second messenger cyclic GMP-AMP (cGAMP) from ATP and GTP (Zhang et al., 2023b). The cytoplasmic DNA may be of exogenous or endogenous origin. Exogenous origin includes pieces of microbial pathogens, and endogenous origin includes genomic DNA damage and mitochondrial breakage (Cao et al., 2020; Chen et al., 2023c). The cGAMP synthesized binds to the adapter protein STING, which is anchored in the endoplasmic reticulum (ER). This binding causes STING to be relocated from the ER to the Golgi apparatus, where it phosphorylates TANK-binding kinase 1 (TBK1) and IκB kinase (Wang et al., 2025). TBK1 subsequently phosphorylates interferon regulatory factor 3 (IRF3), leading to its dimerization and nuclear translocation, which subsequently activates the production of IFN-Is, along with other inflammatory mediators, pro-apoptotic genes, and chemokines (Lam et al., 2021). In addition, IFNs also induce the production of interferon-stimulated genes (ISGs) via the JAK-STAT signaling pathway, which creates a positive feedback loop, thereby activating the cGAS and STING activation (Shaikh et al., 2025). Ultimately, whether localized within the autophagosome or sourced from the Golgi apparatus, STING is transported to the lysosome for degradation (Sliter et al., 2018; Huang et al., 2025c; Yang et al., 2025a).

### Intercellular cGAMP signaling networks

Existing research has revealed that cGAMP is a critical intercellular signaling molecule, playing a key function in intercellular communication and enhancement of immune responses (He et al., 2025). cGAMP transmits intercellularly through two primary pathways: gap junctions and non-specific transmembrane carriers (**Figure 1**). Gap junctions are not only formed between homotypic cell types but also between heterogeneous populations of cells participating in physiological immune responses (Decout et al., 2021; Ge et al., 2025; Tishchenko et al., 2025). Amplification of cGAS–STING activation signals between neurons and between non-neuronal cells and neurons is facilitated by gap junctions, thereby increasing neuroinflammation and immune responses (Elzinga et al., 2022; Ullah et al., 2022). The modulation of cGAS–STING signaling in bystander cells is obtained through inter-neuronal gap junctions, resulting in enhanced neuronal apoptosis (Tan et al., 2022). cGAMP was also discovered to be passed from neurons to microglia, passing neuroinflammatory signals (Ge et al., 2025). Interestingly, gap junction inhibition has the ability to significantly inhibit cGAS–STING-linked gene-mediated neuroinflammation in the brain (Elzinga et al., 2022). Moreover, intercellular cGAMP signaling gap junction-dependently takes place among the cancer cells and plays a critical role in tumor destruction and immune evasion (Yi et al., 2024; Zhang et al., 2024b).

Non-selective transmembrane carriers, or transporters, channels, and pores such as SLC19A1, SLC46A2, P2X7, ABCC1, and volume-regulated anion channels (VRACs), are vital for extruding cGAMP into the extracellular milieu and bringing it into cells (Decout et al., 2021; Blest et al., 2024). Some viruses have been found to target the downregulation of SLC46A2, P2X7, and the VRAC subunits LRRC8A and LRRC8C, thereby limiting innate immune response through decreased intercellular communication mediated by cGAMP (Blest et al., 2024). Ablation of the volume-regulated anion channel LRRC8/VRAC by genetics or chemical inhibition results in impaired cGAMP transfer between tumor-associated macrophages and fibroblasts, decreasing STING-dependent interferon response (Wang et al., 2024a). Furthermore, cGAMP importation by LRRC8C and subsequent STING-p53 activation represents a novel inhibitory mechanism in adaptive immunity and T cells (Concepcion et al., 2022). Some cells possess members of the ectonucleotide pyrophosphatase/phosphodiesterase family that catabolize cGAMP and thus inhibit cGAMP-mediated intercellular communication (Mardjuki et al., 2024).

Vesicles in atypical autophagosomes with activated STING in Rafeesomes are released as R-EVs (RAB22A-induced extracellular vesicles). R-EVs activate the IFNβ pathway in acceptor cells of the tumor microenvironment and thereby enhance anti-tumor immunity (Gao et al., 2022). The transport routes of these new targets must be investigated in future studies on neuroinflammatory and neurodegenerative diseases related to the cGAS–STING pathway to elucidate aberrant intercellular communication and identify new targets for activation.

### Detecting cellular perturbations via the cGAS–STING pathway

The cGAS–STING pathway not only works as a typical pattern recognition receptor but also as a central mediator of danger detection for sensing non-environmentally linked self-DNA (Lei et al., 2023; Zecchini et al., 2023). Intracellular DNA stability can be disturbed by different pathological processes, thereby activating the cGAS–STING pathway (Huang et al., 2023; Xie et al., 2024b; **Figure 1**).

Mitochondrial DNA (mtDNA) is vulnerable to damage induced by reactive oxygen species generated by the electron transport chain in the process of respiration due to its proximity to the electron transport chain complexes (Chen et al., 2007; Green et al., 2008). In contrast to the robust repair machinery that safeguards nuclear DNA, repair pathways for mtDNA are comparatively inefficient, making it more vulnerable to damage (Marx et al., 2025). mtDNA damage is also linked to various neuroinflammatory and neurodegenerative diseases (Gulen et al., 2023; Zong et al., 2024). Within mitochondria, mitochondrial transcription factor A plays a role in stabilizing mtDNA by forming nucleosomes, which is essential for mtDNA stress prevention and blocking the abnormal activation of cGAS (Xie et al., 2024a). Numerous stress signals can induce mitochondrial dysfunction, potentially leading to apoptosis in severe cases, which facilitates the loss of mtDNA compartmentalization (Carloni et al., 2024). Under stress, mtDNA can be expelled through the inner mitochondrial membrane and subsequently released through BAX/BAK or via pores generated by the voltage-dependent anion channel on the outer mitochondrial membrane (Victorelli et al., 2023). The mitochondrial permeability transition pore may provide an alternative route for mtDNA to traverse the inner mitochondrial membrane (Yang et al., 2024a). mtDNA entry into the cytosol activates the cGAS–STING pathway, promoting the upregulated expression of ISGs and the downstream inflammatory and immune responses (Du et al., 2025).

Nuclear DNA can be accessed by cytosolic cGAS through three distinct mechanisms (Peng et al., 2025). The first mechanism is the derepression of retrotransposon elements, specifically long interspersed nuclear element 1 (LINE1). This derepression leads to increased LINE1 transcription and the accumulation of LINE1 cDNA, which activates the cGAS–STING pathway, resulting in the secretion of the senescence-associated secretory phenotype and subsequent cellular senescence (Tang et al., 2024). The second mechanism involves DNA damage or replication stress, which can cause the direct leakage of nuclear DNA into the cytosol (Haase et al., 2022). Furthermore, during senescence, chromatin protrusions may emerge from the primary nucleus, leading to the accumulation of cytosolic chromatin fragments (Dasgupta et al., 2024). In situations of genomic instability, nuclear DNA may be encapsulated into micronuclei during mitosis and later released into the cytoplasm (Zannini et al., 2024). All these mechanisms of cytosolic DNA recognition originating from the nucleus are effectively inhibited by the exonuclease TREX1 (Yang et al., 2025c). Within the cellualr nucleus, nucleosomes and structural chromatin proteins, including histone H1 and the barrier-to-autointegration factor, collaboratively restrict aberrant cGAS activity (Broussard et al., 2023). Notably, these various activation modes are not mutually exclusive; they may redundantly and synergistically trigger cGAS activity in different contexts.

### cGAS–STING pathway and cell death

**Figure 2:**
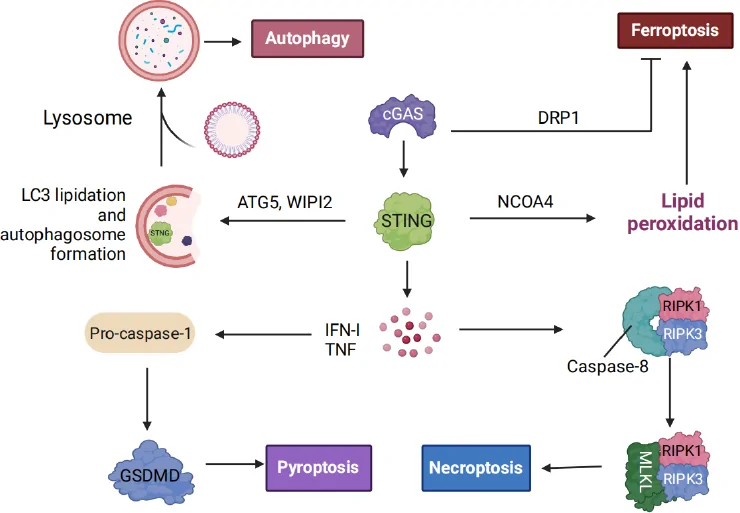
The crosstalk between the cGAS–STING pathway and cell death pathways. The activation of the cGAS–STING pathway can promote various forms of cell death, including autophagy, ferroptosis, pyroptosis, and necroptosis. Upon activation, STING, with the assistance of key proteins such as ATG5 and WIPI2, can bind to LC3 to form phagosomes, which are eventually engulfed by lysosomes. The activation of STING also promotes NCOA4-mediated lipid peroxidation, ultimately leading to ferroptosis. However, the interaction between the cGAS and DRP1 proteins can inhibit the occurrence of ferroptosis. Downstream of the cGAS–STING pathway, IFN-I and TNF facilitate the formation of a complex comprising caspase-8, RIPK3, and RIPK1, which subsequently activates the MLKL, promoting necroptosis. Additionally, IFN-I and TNF can activate pro-caspase-1, leading to the activation of GSDMD and initiating the pyroptosis process. Created with BioRender.com (https://BioRender.com/7hklhao). ATG5: Autophagy-related protein 5; cGAS: cyclic GMP-AMP synthase; DRP1: dynamin-related protein 1; IFN-I: type I interferon; MLKL: mixed lineage kinase domain-like; NCOA4: nuclear receptor coactivator 4; RIPK1: receptor-interacting protein kinase 1; RIPK3: receptor-interacting protein kinase 3; STING: stimulator of interferon genes; TNF: tumor necrosis factor.

Cell death is crucial for tissue and organ development, as well as for removing infected, damaged, or transformed cells to maintain tissue homeostasis (Tsvetkov et al., 2022). Multiple forms of cell death, such as apoptosis, ferroptosis, necroptosis, pyroptosis, autophagy, and immunogenic cell death (Huang et al., 2018; Chen et al., 2025a; Liu et al., 2025a; Zha et al., 2025), are vital in neurological disorders and injuries (Li et al., 2022c; Wang et al., 2022b). Recently, evidence has shown that there is crosstalk between the cGAS–STING pathway and various types of cell death, which in turn affects neuronal survival (**Figure 2**).

#### Autophagy

Autophagy is a crucial cellular process in eukaryotes that entails the sequestration of cytosolic components within double-membraned autophagosomes, which then fuse with endosomal or lysosomal vesicles to facilitate the degradation and recycling of the sequestered substrates (Zhao et al., 2025a). This process consists of five key steps: initiation, nucleation, elongation, autophagosome formation, and degradation (Murthy et al., 2020).

STING contains a domain that interacts with the classical autophagy protein LC3 (Xun et al., 2024). p62 is a member of a broad family of ubiquitin-binding autophagy receptors that link ubiquitin to autophagy through its ubiquitin-binding domain and light chain 3 (LC3)-interaction region. In the initial phase of autophagy, STING dissociates from the ER and is transported to the ER-Golgi intermediate compartment after binding to cGAMP, which supplies membranes for LC3 lipidation and autophagosome formation (Gui et al., 2019). STING is then incorporated into autophagosomes and sorted into lysosomes (Xun et al., 2024). Herein, p62, autophagy protein 5 (ATG5), and WIPI2 are necessary for canonical autophagosome formation stimulated by STING (Wan et al., 2023), while the UNC-51-like autophagy-activating kinase 1 (ULK1) complex and phosphatidylinositol 3-kinase catalytic subunit type 3 (PIK3C3/VPS34) complex are dispensable (Gui et al., 2019). RAB7A facilitates the trafficking of STING to lysosomes (Wang et al., 2023c), and STING is then degraded in lysosomes. Activation of the LKB1/AMPK/ULK1 pathway can promote STING-dependent autophagy, alleviating oxidative stress-induced damage (Liu et al., 2025b). Rong et al. (2022) reported that energy stress disrupts the STING-STX17 interaction, resulting in the activation of autophagy mediated by STX17.

cGAS can directly bind to Beclin-1 to induce autophagy in a STING activation-independent manner (Liang et al., 2014). Rubicon functions as a negative regulator of autophagy through binding to the Beclin-1-PI3KC3 complex and inhibiting PI3KC3 activity (Liu et al., 2024b). dsDNA binding to cGAS enhances its binding to Beclin-1, which reduces Rubicon binding to Beclin-1. The reduction causes the release of Rubicon from the Beclin-1-PI3KC3 complex, which ultimately activates the PI3KC3 kinase and triggers autophagy (Liu et al., 2024b). Besides, ATG4B can initiate autophagic degradation by targeting TBK1 (Xie et al., 2023a). In summary, cGAS–STING-related genes can activate autophagy through multiple pathways. Further studies are needed on the roles of autophagy pathways participating in the cGAS–STING pathway in neurological diseases and injuries.

#### Necroptosis

Necroptosis is a regulated form of cell death characterized by membrane rupture and the release of cellular contents (Jeong et al., 2025). It is typically triggered by extracellular or intracellular stress signals and involves the activation of receptor-interacting protein kinases (RIPK1 and RIPK3), which then interacts with and phosphorylates RIPK3 to form the necrosome. The necrosome phosphorylates lineage kinase domain-like protein (MLKL), leading to membrane permeabilization and cell lysis (Chen et al., 2025d). It is involved in various diseases including neurodegenerative and neuroinflammatory disorders (Ni et al., 2024; Huang et al., 2025d).

The IFN signaling pathway is crucial for triggering necroptosis (Grilo Ruivo et al., 2025). DNA from DNA damage repair or mitochondrial stress activates the cGAS–STING pathway to yield IFNs and tumor necrosis factor-α (Wang et al., 2024c). These cytokines subsequently activate RIPK1 and RIPK3, as well as MLKL, ultimately causing necroptosis (Dwamena et al., 2024). By binding to receptors and activating STAT1/2 transcription factors, IFNs sustain the expression of various ISGs, including MLKL (Sarhan et al., 2019). Targeted inhibition of cGAS or STING activation can suppress necroptosis (Zhu et al., 2024a). MRE11, which serves as a sensor for DNA double-strand breaks, plays a pivotal role in regulating cGAS activation (Técher et al., 2024). MRE11–RAD50–NBN complex binding to nucleosome fragments relieves the bound cGAS, thereby promoting necroptosis through the Z-DNA binding protein 1-RIPK3-MLKL pathway (Cho et al., 2024).

#### Ferroptosis

Ferroptosis is an iron-dependent form of regulated cell death distinct from apoptosis, necrosis, and autophagy. It is mostly brought about by the accumulation of lipid peroxides to toxic levels, typically due to suppression of the glutathione-dependent antioxidant pathway or suppression of glutathione peroxidase 4 (GPX4) (Zhou et al., 2025a). Ferroptosis has been implicated in numerous physiological and pathological processes, such as neurodegeneration, ischemia-reperfusion injury, and tumors (Fan et al., 2025a; Zheng et al., 2025).

Loss of GPX4 activity directly or indirectly is presently known as a key event initiating ferroptosis (Aishajiang et al., 2025). GPX4 works synergistically with glutathione to reduce free hydrogen peroxide and organic peroxides to water or to their corresponding alcohols (Wang et al., 2023d). With its deficiency, GPX4 becomes inactivated and undergoes increased lipid peroxidation. Recent studies have shown that GPX4 is crucial in initiating the cGAS–STING pathway (Jiang et al., 2025). GPX4 deficiency elevates lipid peroxidation, which carbonylates STING and suppresses its localization from the endoplasmic reticulum to the Golgi complex, and thus inhibits the immune response (Deng et al., 2023; Aishajiang et al., 2024). However, Chen et al. (2025c) confirmed that stress induced by lipotoxicity elevated the expression of STING through downregulation of GPX4 expression and enhanced lipid peroxidation. Interestingly, the application of STING inhibitors is able to suppress ferroptosis caused by lipotoxicity. STING inhibition can suppress nuclear receptor coactivator 4-induced ferritinophagy, which is tasked with preventing ferroptosis (Li et al., 2023). Research has shown that cGAS anchors to the outer mitochondrial membrane and binds to dynamin-related protein 1 to facilitate its oligomerization (Chen et al., 2023b). In the absence of cGAS or dynamin-related protein 1 oligomerization, there is an accumulation of mitochondrial reactive oxygen species (ROS), results in augmented ferroptosis (Li et al., 2023).

#### Pyroptosis

Pyroptosis is an inflammatory cell death mechanism triggered by inflammasomes, which are large multi-protein complexes that include nucleotide-binding oligomerization domain-like receptors (NLRs) such as NLRP3, NLRC4, and AIM2, as well as the adapter protein ASC and pro-caspase-1 (Li et al., 2024a; Lin et al., 2025b; Yang et al., 2025b). This process leads to the oligomerization of gasdermin D, which forms pores in the plasma membrane, causing water influx, cell lysis, and subsequent cell death (Xiao et al., 2023).

The IFN response mediated by the cGAS–STING pathway upregulates the expression of caspase-1 and caspase-11, promoting increased secretion of interleukin (IL)-1β and promoting pyroptosis (Liu et al., 2022). Gasdermin D has been identified as a trigger for rapid mitochondrial collapse and the initial cytosolic accumulation of mtDNA, which facilitates the release of mtDNA from cells upon plasma membrane rupture, further activating the cGAS-STING pathway (Yang et al., 2025; Yu et al., 2025b). In canonical inflammasome activation, caspase-1 serves to restrict cytosolic DNA-mediated cGAS–STING activation through the direct cleavage of cGAS, thereby acting as a negative feedback mechanism (Ding et al., 2022).

## A Bibliometric Analysis of the Role of the cGAS–STING Pathway in Neurological Disorders

CiteSpace (6.4.R1 Advanced) is the primary tool of bibliometric visualization to predict the role of the cGAS–STING pathway in neurological disorders/injuries. The core collection of the Web of Science is used as the literature source. Then search strategies are formulated for the role of the cGAS–STING pathway in neurological disorders/injuries: (ALL=(cGAS–STING pathway) AND ALL=(neurological disorders); ALL=(cGAS–STING pathway) AND ALL=(nerve injuries); ALL=(cGAS–STING pathway) AND ALL=(neuroinflammation); ALL=(cGAS–STING pathway) AND ALL=(tissue engineering); ALL=(cGAS–STING pathway) AND ALL=(nervous system); ALL=(cGAS–STING pathway) AND ALL=(neurodegeneration)). The type of literature is limited to “article” or “review,” the language is limited to English, and the search is restricted to the Web of Science Core Collection for the period from January 1, 2001 to January 11, 2025. After eliminating the duplicate literature, a total of 224 articles were obtained. The comprehensive visualization method, including co-citation reference analysis, and keyword co-occurrence analysis, is applied to analyze the included literature.

**Figure 3:**
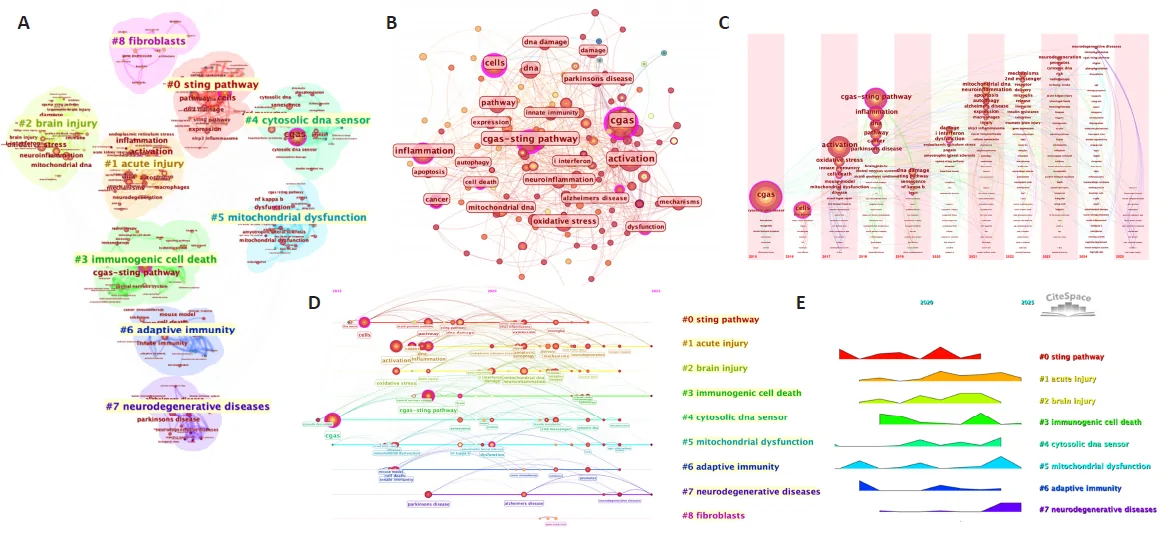
Analysis of co-occurrence keywords. (A) Keywords cluster analysis. (B) The visualization map of co-occurrence keywords. The genes associated with the cGAS–STING pathway exhibit significant correlations with neuroinflammation, cGAS–STING pathway, Alzheimer’s disease, Parkinson’s disease, and cell death. (C) Time zone map of clusters. (D) Timeline map of clusters. (E) Landscape of clusters. cGAS: Cyclic GMP-AMP synthase; STING: stimulator of interferon genes.

A clustering analysis of the co-occurring keywords was conducted (**Figure 3A**). Nine clusters were produced, i.e., “STING pathway,” “acute injury,” “brain injury,” “immunogenic cell death,” “mitochondrial dysfunction,” “adaptive immunity,” “neurodegenerative disease,” and “fibroblasts.” These findings confirm a high correlation between the cGAS–STING pathway and neurological diseases and injuries. The keywords in this study encompass ‘neuroinflammation,’ ‘cGAS–STING pathway,’ ‘Alzheimer’s disease,’ ‘Parkinson’s disease,’ and ‘cell death,’ among others (**Figure 3B**). When considered alongside the timeline map and time zone map of clusters, it can be noted that the contribution of the cGAS–STING pathway to “immunogenic cell death,” “mitochondrial dysfunction,” and “neurodegenerative disease” is generating increasing attention (**Figure 3C–E**).

**Figure 4:**
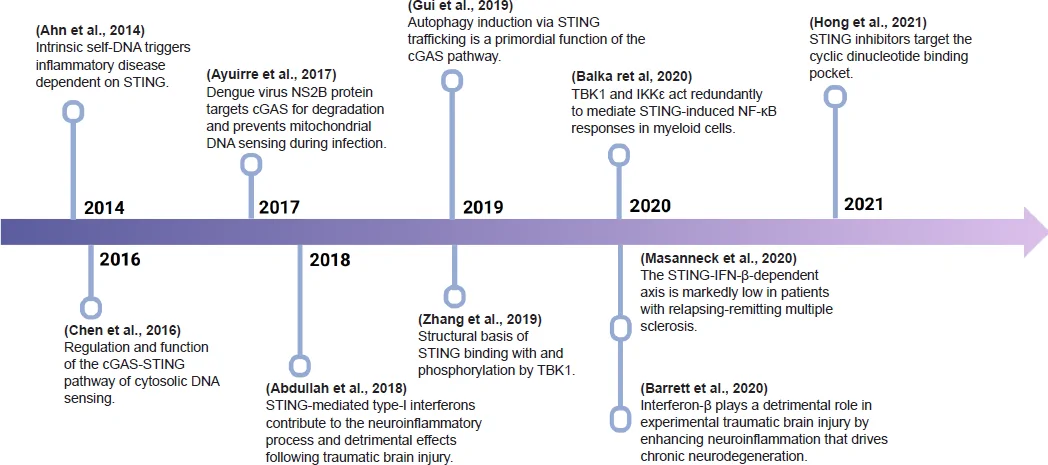
Milestone events in research related to cGAS–STING pathway and neurological diseases. Using centrality scores based on the SVA algorithm, we have identified and sorted in chronological order the milestone events in research related to the “cGAS–STING pathway and neurological diseases/injuries” field. Created with BioRender.com (https://BioRender.com/m74n213). cGAS: Cyclic GMP-AMP synthase; STING: stimulator of interferon genes.

There has been a notable increase in recent studies investigating the role of the cGAS–STING pathway in neurological disorders/injuries. We have given an overview of the milestone events in research related to the cGAS–STING pathway and neurological diseases and injuries, according to the literature centrality scores provided by the SVA algorithm (**Figure 4** and **Table 1**). In the early stages of research between 2014 and 2017, most studies focused on elucidating the mechanism of the cGAS–STING pathway senses intracellular DNA and the downstream immune and inflammatory responses (Ahn et al., 2014; Chen et al., 2016; Aguirre et al., 2017). With the progress of research, the cGAS–STING pathway and downstream IFN-I signaling are shown to be involved in neuroinflammation-mediated secondary injury following traumatic brain injury (TBI). The functions of key proteins within the cGAS–STING pathway, such as TBK1 and IRF3, in mediating neuroinflammation have been elucidated (Gui et al., 2019; Zhang et al., 2019; Balka et al., 2020). The past five years have seen greater investigation of the role of the cGAS–STING pathway in other neuroinflammatory disorders and neurodegenerative disorders have expanded, underscoring its regulatory function across a broader spectrum of neurological diseases and injuries (Barrett et al., 2020; Masanneck et al., 2020). Besides, cGAS–STING pathway inhibitors have demonstrated strong efficacy in preventing the onset of inflammation and autoimmune disease, hence enabling subsequent recovery of neurofunction (Hong et al., 2021).

**Table 1:** The top ten most highly cited references ranked by the centrality

Number	Author	Article title	Publication year	Centrality
1	Aguirre et al.	Dengue virus NS2B protein targets cG AS for degradation and prevents mitochondrial DNA sensing during infection	2017	0.34
2	Ahn et al.	Intrinsic self-DNA triggers inflammatory disease dependent on STING	2014	0.28
3	Abdullah et al.	STING-mediated type-I interferons contribute to the neuroinflammatory process and detrimental effects following traumatic brain injury	2018	0.22
4	Hong et al.	STING inhibitors target the cyclic dinucleotide binding pocket	2021	0.08
5	Barrett et al.	Interferon-β plays a detrimental role in experimental traumatic brain injury by enhancing neuroinflammation that drives chronic neurodegeneration	2020	0.07
6	Gui et al.	Autophagy induction via STING trafficking is a primordial function of the cGAS pathway	2019	0.07
7	Masanneck et al.	The STING-IFN-β-dependent axis is markedly low in patients with relapsing-remitting multiple sclerosis	2020	0.07
8	Zhang et al.	Structural basis of STING binding with and phosphorylation by TBK1	2019	0.07
9	Balka et al.	TBK1 and IKKε act redundandly to mediate STING-induced NF-kB responses inmyeloid cells	2020	0.06
10	Chen et al.	Regulation and function of the cGAS-STING pathway of cytosolic DNA sensing	2016	0.06

cGAS: Cyclic GMP-AMP synthase; IFN: interferon; NF-κB: nuclear factor kappa B; STING: stimulator of interferon genes.

In summary, the bibliometric analysis highlights the growing attention on the role of the cGAS–STING pathway in neurological injuries and diseases. Activation of this pathway plays a critical role in both neurological injuries and neurodegenerative disorders (e.g., Alzheimer’s disease and Parkinson’s disease). Notably, the regulation of cGAS–STING-related genes has emerged as a key research focus due to its potential protective effects in promoting neural repair and regeneration.

## Regulation of cGAS–STING Signaling on Neurological Disorders

**Figure 5:**
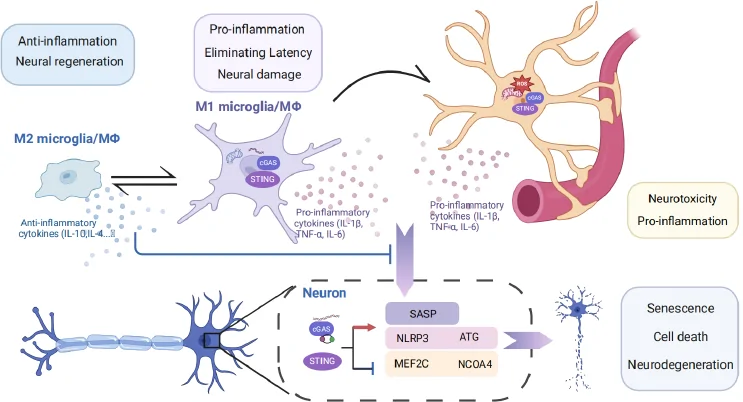
Schematic diagram illustrating the role of the cGAS–STING pathway in the nervous system. The activation of cGAS–STING-related genes promotes M1 phenotype in macrophages/microglia, enhancing the release of pro-inflammatory cytokines by these cells as well as by astrocytes. Subsequently, these cytokines act on neurons, activating the expression of the cGAS–STING pathway and promoting the downstream expression of genes such as NLRP3, ATG, MEF2C, and SASP, leading to cellular senescence, death, and neurodegeneration. This effect can be reversed by inhibiting the M1 phenotype of macrophages/microglia. Created with BioRender.com (https://BioRender.com/3h99n7l). ATG: Autophagy-related gene; cGAS: cyclic GMP-AMP synthase; MEF2C: myocyte enhancer factor 2C; NLRP3: nucleotide-binding oligomerization domain-like receptor protein 3; SASP: senescence-associated secretory phenotype; STING: stimulator of interferon genes.

cGAS–STING-related genes exert a considerable influence on the behavioral patterns and cellular fates of both neuronal and non-neuronal cells in the nervous system. These genes specifically regulate the functions of glial cells, which provide structural support and nutritional sustenance to neurons (Wang et al., 2023e; Hertzog et al., 2025), as well as microglia and macrophages, which are essential for immune surveillance and inflammation in the nervous system (Morozzi et al., 2021). As a result, disruptions in the expression or activity of cGAS–STING-related genes can significantly impact numerous neuropathological processes, including the initiation and progression of neuroinflammation, neurodegenerative disorders, aging, and cell death regulation (Gamdzyk et al., 2020; Gulen et al., 2023; **Figure 5**).

### Nervous system injury

Nervous system injury refers to a pathological process wherein structural or functional impairment of the nervous system, induced by trauma, ischemia, intoxication, or other pathological factors, precipitates a cascade of motor, sensory, cognitive, or autonomic dysfunction (Zhu et al., 2024a). Broadly, this injury can be categorized into central nervous system injury and peripheral nerve injury. The pathophysiological core involves primary mechanical/ischemic insults triggering inflammatory responses, ionic dysregulation, and neuronal apoptosis, followed by secondary glial scar formation, neurotransmitter imbalance, and axonal demyelination (Hilton et al., 2024). The cGAS–STING pathway exerts a pivotal role in orchestrating and modulating microglia/macrophage polarization and neuronal viability (Kong et al., 2024; Wang et al., 2024a). Administration of STING inhibitors has demonstrated a promising efficacy in mitigating neuroinflammation and facilitating functional recovery post-neural injury, highlighting a novel therapeutic avenue for mitigating secondary damage and promoting nerve regeneration (Wang et al., 2023a).

#### Peripheral nerve injury

After peripheral nerve injury (PNI), a cascade of pathophysiological events unfolds in the damaged nerves, leading to proximal axonal disruption, neuronal stress responses, and Wallerian degeneration in the distal stump (Waller, 1851). During the first 0–3 days following the injury, Schwann cells (SCs) in the injured region dedifferentiate into repair SCs (rSCs) and release chemokines to recruit inflammatory cells, including T lymphocytes and neutrophils (Berner et al., 2022). Simultaneously, macrophages activate, initiating the immune response, clearing myelin debris, and the secretion of VEGF-A and HIF-1α, which triggers by the perception of hypoxia. rSCs and M1 macrophages collaborate to clear myelin debris (Sun et al., 2024b). Angiogenic endothelial cells increase Plexin-D1 expression, guiding blood vessel migration toward the distal end and establishing pioneering pathways (Bhat et al., 2024). By 1–2 weeks post-injury, rSCs align with newly formed blood vessels to facilitate axon regrowth, while M2 macrophages secrete neurotrophic factors (Cattin et al., 2015). Four weeks after PNI, inflammation diminishes, and the recruited inflammatory cells withdraw. The local area then retains resident macrophages, which exist in a mixed state (Sprenger-Svačina et al., 2024). rSCs subsequently differentiate back into their mature state, remyelinate the regrown axon, and restore neural electrical conduction (Wan et al., 2024).

*Neurons and Schwann cells*

Following PNI, neurons at the injury site secrete IFNγ, which activates the JAK-STAT pathway through the IFNGR receptors on SCs, leading to the expression of ISGs (Wang et al., 2023e). Subsequently, the expression of cGAS in SCs results in the production and secretion of cGAMP, which acts on neurons in a paracrine manner. cGAMP activates the cGAS–STING pathway in neurons, promoting the formation and expansion of growth cones and enhancing the expression of regeneration-associated genes, ultimately facilitating axon elongation and peripheral nerve regeneration (PNR) (Wang et al., 2023e). The inhibition of STING reduced the expression of regeneration-associated genes, including Sprr1a, Gadd45a, and Tubb6, thereby suppressing axonal elongation. Notably, Tubb6 knockdown blocked the axon elongation effect of cGAMP, suggesting that Tubb6 may serve as a functional downstream effector of STING activation in neurons. Furthermore, the STING agonist increased the growth rate of microtubules by enlarging the growth cone area of axons and recruiting more EB1-GFP (a marker for growing microtubules) (Quigley et al., 2024) to the growth cone. Moreover, activation of ISGs in neural crest stem cells might impede the differentiation of SCs and the subsequent formation of myelin (Gacem et al., 2020; Bedoui et al., 2022). However, the precise mechanisms by which ISGs influence SCs and neurons remain to be fully elucidated.

*Macrophage and immune response*

IFNγ is recognized as a prototypical activator of M1 macrophage phenotypes (Ge et al., 2022). The mitochondrial DNA-cGAS–STING signaling pathway plays a crucial role in facilitating the polarization of macrophages toward the M1-like phenotype (Geng et al., 2023; **Figure 5**). Inhibition of cGAS and STING gene activation leads to a significant reduction in the phosphorylation levels of TBK1 and IRF3 within macrophages (Chen et al., 2025e). This reduction subsequently results in decreased mRNA expression levels of IFN-α and IFN-β, thereby impairing the polarization of macrophages toward the M1 phenotype (Geng et al., 2023; Wang et al., 2024e). Furthermore, the activation of the JAK1/2-STAT1 signaling pathways is recognized as a regulatory mechanism that modulates macrophage polarization toward the M1 type, and this modulation can be inhibited by STAT1 inhibitors (Sun et al., 2022b). Shen et al. (2023) found that mtDNA in the cytosol could activate cGAS signaling and induce macrophages to polarize into the M1 phenotype via the mTORC1 pathway in response to lipopolysaccharide attack, which can be reversed by a mTORC1 pathway inhibitor. Intriguingly, Morozzi et al. (2021) reported a decrease in *CSF1* gene expression in the sciatic nerve of STING knockout mice, noting that pharmacological inhibition of CSF1R results in a reduction of M1 macrophages. Treatment with STING inhibitors promoted M2 macrophage polarization, reduced M1 polarization, and suppressed the expression of pro-inflammatory factors (Su et al., 2023). However, how this process affects M2 macrophage behavior following PNI remains to be explored.

It is critical to recognize that immune cells outside macrophages also have important roles to play in the regulation of PNI. The polarization of macrophages is affected by the interaction between T helper (Th) cells and regulatory T cells (Tregs) (Choi et al., 2024; Zhang et al., 2024a). Activation of the cGAS–STING pathway amplifies Th cell and Treg activation, recruitment, and phenotypic switching (Field et al., 2020; Benoit-Lizon et al., 2022; Choi et al., 2024). In naive CD4 T cells, the STING gene activation is controlled through the IRF3/IFN-β/IFNAR axis, which controls IFN-γ release from Th1 cells and activates mTOR signaling and IL-9 production in Th9 cells (Benoit-Lizon et al., 2022). Since Th1 cytokines can polarize macrophages towards the M1 subtype, the activation of the STING gene in Th cells may promote the transition of macrophages to the M1 phenotype, modulating PNI. Conversely, Tregs primarily exert their actions to suppress undue immune responses and maintain immune tolerance (Fusco et al., 2025). Activation of the mtDNA-cGAS–STING pathway in Treg cells induces IL-10 secretion to reduce tissue damage because of uncontrolled inflammation or autoimmune attack (Field et al., 2020).

#### Central nervous system injuries

Injuries to the central nervous system (CNS), including TBI and spinal cord injury (SCI), initiate a complex cascade of biological responses that lead to cellular loss, axonal degeneration, and limited regenerative capacity. The pathophysiological processes following CNS trauma can be categorized into two interconnected phases: primary and secondary injuries (Tsai et al., 2024). The primary injury is characterized by localized contusions, diffuse axonal injury, hematomas, and swelling. In contrast, secondary injury commences hours to months post-initial trauma. It encompasses inflammatory reactions, oxidative stress, excitotoxicity, apoptosis, and necrosis, all of which exacerbate the primary injury (Yu et al., 2025a). Notably, the activation of the cGAS–STING pathway predominantly exerts a crucial regulatory function in secondary injury (Jiang et al., 2021).

*Traumatic brain injury*

The neuropathological mechanisms associated with TBI are complex and multifaceted. Prominent pathological features encompass a range of conditions, including persistent neuroinflammation, tauopathy, pathological changes involving amyloid-beta (Aβ) and TAR DNA-binding protein 43 (TDP-43), axonal degeneration, white matter degradation, and neuronal loss (Hay et al., 2016; Lai et al., 2024). Studies indicate that chronic and persistent neuroinflammatory responses following TBI significantly contribute to secondary neuronal damage and hinder neural repair processes (Abdullah et al., 2018).

TBI-induced mitochondrial stress results in the release of mtDNA, which stimulates the cGAS–STING pathway. This activation initiates downstream signaling involving TBK1 and IRF3, leading to the expression of IFN-I and proinflammatory cytokines (Fritsch et al., 2022). The cGAS–STING pathway is engaged in post-TBI neuroinflammation, and targeting cGAS–STING-related genes has demonstrated efficacy in reducing neuroinflammation and improving patient prognosis (Barrett et al., 2021). When knocking out the STING gene in microglia, researchers observed reduced cortical lesion volume and cell death, making late-stage neurofunctional recovery possible (Fritsch et al., 2024). Fritsch et al. (2022) found that the immune modulator NLRX1 is capable of sequestering STING and inhibiting its downstream activation, thereby minimizing neuroinflammation-associated damage. Moreover, the cGAS–STING pathway is involved in various forms of cell death following TBI, as its activation is known to mediate autophagy and ferroptosis, which contribute to neuronal death and exacerbate neurological damage (Hu et al., 2022; Kumari et al., 2024). Studies have also indicated that central and peripheral immune cells react collectively to brain injury and cooperate in neuroinflammation, although the precise mechanisms underpinning this are still to be revealed (Fritsch et al., 2024).

*Spinal cord injury*

The pathology of SCI is primarily dealing with the local effect zone, where a cascade of primary injuries occurs within the spinal cord as a result of external forces (Zipser et al., 2022). Initial manifestations include hemorrhage in the central gray matter, edema in the white matter, and necrosis of neurons and glial cells. In the phase of acute inflammatory response, neutrophils, and microglia rapidly infiltrate the injured area, clearing necrotic cell debris and initiating tissue repair processes (Wei et al., 2024). Afterwards, astrocytes become activated and proliferate, forming a glial scar to limit damage propagation on one hand, and secreting a multitude of growth factors and cytokines to establish microenvironmental support for the subsequent regeneration process on the other (Quan et al., 2025). Oligodendrocyte precursor cells, in response to injury signals, migrate to the damaged region and differentiate into mature oligodendrocytes, participating in the repair and regeneration of myelin (Fan et al., 2025a). The typical manifestations of secondary injury after SCI are excessive amounts of reactive oxygen species, which amplify lipid peroxidation of the cell membrane (Li et al., 2025c). This process alters the structure and function of the membrane and interferes with the function of some crucial enzyme systems, including Na^+^, K^+^-ATPase, adenylate cyclase, and cytochrome oxidase (Li et al., 2020b; Qian et al., 2024).

The cGAS–STING pathway also plays a role in modulating neuroinflammation during the secondary injury phase following SCI by managing the M1/M2 phenotypic polarization of microglia (Brennan et al., 2022). Wei et al. (2024) observed that the downregulation of mitofusin-2 (Mfn2) in microglia after SCI leads to an imbalance between mitochondrial fusion and fission, leading to mtDNA release. This release activates STING, initiating an inflammatory response that promotes microglial polarization towards M1 phenotype, thereby exacerbating inflammatory injury. Meanwhile, targeting and inhibition of the cGAS/STING/NF-κB pathway can promote microglial polarization from an M1 to an M2 phenotype after SCI, inhibiting neuronal death and neuropathic pain, and improving tissue repair and functional recovery (Wu et al., 2022; Fan et al., 2025b). It has also been shown that autophagy impairment in microglia and macrophages following SCI exacerbate the activation of the cGAS–STING pathway, leading to increased production of pro-inflammatory cytokines (Li et al., 2022b). The cGAS–STING pathway therefore plays a crucial role in mediating inflammation-driven injury following SCI, and inhibition of cGAS–STING-related genes may be a promising therapeutic strategy to promote neural repair in SCI.

*Hypoxic-ischemic encephalopathy*

Hypoxic-ischemic encephalopathy (HIE) is a complex pathophysiological cascade initiated by diminished cerebral blood flow and oxygenation (Ji et al., 2022). The root characteristic of the condition is the initial exhaustion of energy, with hypoxia disrupting mitochondrial oxidative phosphorylation to result in decreased ATP production but stimulate anaerobic glycolysis and lactate accumulation (Rodríguez et al., 2020). This is then succeeded by the hypersecretion of excitatory amino acids, predominantly glutamate, which leads to the overactivation of NMDA receptors. Overactivation of NMDA receptors leads to influx of calcium and intracellular calcium overload, which ultimately leads to injury of the neurons through excitotoxicity (Zhang et al., 2023c). After ischemic reperfusion, microglial activation with stronger activated oxidative stress hastens the inflammatory cascade, leading to enhanced neuronal death and brain injury (Li et al., 2025d).

The cGAS–STING pathway activation is implicated not only in mediating inflammatory damage in HIE but also in cellular death processes. Research indicates that silencing STING via small interfering RNA (siRNA) in HIE rat models results in reduced infarct size, mitigated cortical neurodegenerative changes, and enhanced neurobehavioral outcomes (Gamdzyk et al., 2020). Moreover, STING co-localizes with the lysosomal marker LAMP-1, and blocking LINE-1 retrotransposons—potential upstream activators of the cGAS–STING pathway—effectively inhibits STING-mediated inflammation while decreasing the expression of cathepsin B and Bax, as well as the cleavage of caspase-3 (Gamdzyk et al., 2020). Additionally, inhibiting cGAS not only downregulates downstream STING expression but also significantly diminishes the production of NLRP3 inflammasomes and microglial pyroptosis (Shen et al., 2024). In summary, targeting STING-mediated inflammatory damage and cellular death may facilitate neurorepair following HIE.

### Cerebrovascular disease

#### Ischemic stroke

During ischemic events, oxidative damage makes cytosolic DNA release from mitochondria and/or the nucleus, and dying neurons release extracellular DNA. These DNA fragments are recognized by cytoplasmic cGAS. These DNA fragments can be recognized by cGAS in the cytoplasm. Additionally, recent research has demonstrated that histone deacetylase 3 expression is upregulated in microglia following ischemic stroke, facilitating the nuclear localization of p65 through deacetylation at lysine 122, which enhances cGAS transcription (Liao et al., 2020). Collectively, these mechanisms activate the cGAS–STING pathway, driving neuroinflammatory responses (Kong et al., 2024).

The cGAS–STING pathway regulates neural regeneration by modulating microglial phagocytic activity after ischemic stroke. The activation of the cGAS–STING pathway significantly obstructs the clearance of myelin debris, which is due to impaired microglial capabilities in the uptake and degradation of myelin debris, as well as lipid transport (Maimaiti et al., 2024). A possible mechanism for this impairment is the disruption of lipid transport functions caused by the activation of cGAS–STING-related genes. Additionally, Wu et al. (2024) inhibiting STING can reduce microglial phagocytic capacity for synapses around the infarct area, thereby alleviating stroke-induced motor dysfunction.

Following stroke, pyroptosis occurs in the penumbral region of the brain. The administration of STING inhibitors can significantly reduce microglial-mediated neuroinflammatory responses and neuronal pyroptosis (Deng et al., 2024a). The underlying mechanism may involve the activation of NLRP3-mediated caspase-1 (Li et al., 2020a). Specifically, the activation of STING leads to the subsequent activation of TBK1 and NF-κB, which in turn activates NLRP3. NLRP3 then targets and cleaves caspase-1. Activated caspase-1 triggers the cleavage of gasdermin D and IL-1β, ultimately initiating pyroptosis and neuroinflammation.

In cerebral ischemia-reperfusion injury resulting from stroke, neuronal autophagy may lead to excessive ferritinophagy and iron deposition (Li et al., 2021). This process significantly downregulates the expression of GPX 4 and nuclear receptor coactivator 4, causing abnormal ferritin accumulation in ischemic brain tissue (Hanke and Rami, 2022). By inhibiting STING, nuclear receptor coactivator 4-mediated ferritinophagy can be reduced, which simultaneously decreases oxidative stress, ferroptosis, autophagy, apoptosis, and infarct volume, ultimately alleviating cerebral ischemia-reperfusion injury after stroke (Li et al., 2023). Additionally, Liu et al. (2025b) demonstrated that activation of the LKB1/AMPK/ULK1 pathway enhances the autophagy-dependent degradation of STING, thereby mitigating mitochondrial damage and oxidative stress, and protecting neurons from apoptosis in cerebral microvascular endothelial cells following ischemia/reperfusion injury.

Following ischemic stroke, a notable influx of peripheral neutrophils is observed at the site of cerebral lesions, where they release neutrophil extracellular traps (Denorme et al., 2022). Inhibitors of peptidylarginine deiminase 4 can inhibit the cGAS–STING pathway by reducing neutrophil extracellular trap production in ischemic brain tissue. Consequently, this inhibition promotes microglial polarization towards an anti-inflammatory M2-like phenotype and mitigates ischemic and reperfusion injury (Sun et al., 2023a).

#### Cerebral venous sinus thrombosis

Cerebral venous and sinus thrombosis is a rare cerebrovascular condition caused by blood clot formation in the brain’s venous sinuses, leading to impaired venous drainage and increased intracranial pressure. This condition commonly presents with symptoms such as severe headaches, seizures, and focal neurological deficits (Furie et al., 2021; Burn et al., 2022).

Cerebral venous and sinus thrombosis triggers the release of dsDNA into the cytoplasm, initiating an inflammatory response through the activation of the cGAS–STING pathway. Inhibition of cGAS activation results in decreased levels of inflammatory cytokines, while also suppressing the expression of the NLRP3 inflammasome and components related to pyroptosis. Furthermore, treatment with cGAS inhibitors alleviates oxidative stress, reduces the number of microglia and neutrophils, and improves neuronal apoptosis, degeneration, and neurological deficits following cerebral venous and sinus thrombosis (Ding et al., 2022).

### Neurodegenerative diseases

Neurodegenerative diseases are a group of chronic progressive neurological disorders, primarily characterized by the gradual loss of neuronal structure and function (Trejo-Lopez et al., 2022). These diseases are typically accompanied by symptoms such as cognitive decline and movement disorders. Recent investigations have brought the cGAS–STING pathway into sharp focus, identifying it as a pivotal regulator of neuroinflammation in neurodegenerative contexts.

#### Alzheimer’s disease

Alzheimer’s disease (AD) is a progressive degenerative disorder of the central nervous system dominated by worsening cognitive performance and behavioral issues. Research has emphasized the crucial function of the innate immune response in the pathogenesis of AD (He et al., 2023). This condition is marked by Aβ deposition the deposition of Aβ and the formation of neurofibrillary tangles comprised of hyperphosphorylated and misfolded tau proteins in the brain, along with neuroinflammation (van Olst et al., 2025). Microglia at the beginning of AD are protective and regulate their phagocytic activity moderately to eliminate Aβ deposits. But with the disease at its late stages, there is an aberrant accumulation of Aβ that triggers overactivation of microglia, resulting in high levels of pro-inflammatory cytokine production and dysfunction in Aβ clearance.

Inhibition of STING expression can alleviate microglia-dependent neuroinflammatory injury, rescue cellular senescence, and restore the Aβ phagocytic function of microglia, and therefore decelerate neurodegeneration progression (Yuan et al., 2024). Conditional cGAS ablation in microglia not only limits Aβ deposition but also significantly reduces neuroinflammation and neuronal damage, enabling subsequent restoration of cognitive functions (He et al., 2023). Loss of cGAS in microglia also lowers levels of dystrophic neurites and thereby preserves synaptic integrity and neuronal function. Previous studies have proven the occurrence of mitophagy in AD, in which DNA that is released to the cytosol during autophagy triggers the abnormal stimulation of the cGAS–STING pathway (Xie et al., 2023b). Nicotinamide adenine dinucleotide (NAD^+^) is a vital human cell metabolite, implicated in processes such as DNA repair and mitophagy (Campbell, 2022). In the study conducted by Hou et al. (2021), treatment with nicotinamide riboside, a precursor of NAD^+^, resulted in reduced DNA release during mitophagy, thus preventing the activation of the cGAS–STING signaling pathway in microglia. Udeochu et al. (2023) demonstrated that the activation of cGAS in microglia disrupts the neuronal transcriptional network regulated by myocyte enhancer factor 2C (MEF2C) through IFN-I signaling, ultimately leading to a reduction in cognitive flexibility. cGAS deletion by genetics or drug-mediated inhibition decreases pathogenic tau protein-induced neurotoxicity and restores synaptic integrity, plasticity, and memory by preventing the cGAS-IFN-MEF2C axis (Udeochu et al., 2023). Moreover, activation of the cGAS–STING pathway could influence the function of other glial cells in AD. Chemerin is a neuroinflammatory adipokine that binds to its receptor chemokine-like receptor 1 (CMKLR1), which is also an Aβ receptor (Chen et al., 2022c). Research by Liu et al. (2024c) has indicated that exogenous chemerin activates the CMKLR1/STING signaling pathway, thereby promoting astrocyte migration and decreasing the accumulation and recruitment of Aβ.

The mtDNA-cGAS–STING pathway mediates neutrophil infiltration of the brain tissue through the NLRP3/IL-1β axis. Knockdown of this pathway is effective at preventing neuronal apoptosis and increasing cognitive impairment (Xia et al., 2024). In addition, c-Jun protein overexpression, responsible for Aβ-induced neurodegeneration, results in RNA-DNA hybrid cytoplasmic accumulation, thereby triggering apoptosis via the cGAS–STING pathway. Consequently, elevated caspase-3 levels trigger programmed cell death in neurons and precursor cells of AD patients (Scopa et al., 2023).

In total, the cGAS–STING pathway plays a multifaceted and dynamic role in AD. It participates in the clearance of pathogenic tau proteins, Aβ plaques, and dead cells by glial cells but, simultaneously, triggers and exacerbates neuroinflammation and progresses AD. These varied responses may correlate with different stages of the disease. Different cell types respond uniquely to cGAS–STING activation, and there is complex crosstalk among such cells. Greater research effort must be applied to delineate such complex mechanisms, which will allow for more effective therapy.

#### Amyotrophic lateral sclerosis

Amyotrophic lateral sclerosis (ALS) is a progressive neurodegenerative disease that is marked by the degeneration of upper and lower motor neurons (Álvarez-Sánchez et al., 2025). ALS clinically presents with muscle weakness and atrophy, which gradually advances to the limbs, trunk, and laryngeal-facial regions, eventually causing respiratory dysfunction in advanced stages (Itou et al., 2024). cGAS–STING pathway activation has been found in postmortem motor neurons of patients with ALS (Marques et al., 2024), and demonstrates a close link between cGAS–STING pathway activation and ALS pathogenesis. SOD1 and TDP-43 mislocalization to mitochondria leads to mitochondrial toxicity and excessive production of ROS (Yu et al., 2020). This mechanism generates mtDNA leakage, that activates neighboring astrocytes and microglia, next cGAS–STING-dependent upregulation of NF-κB and IFN-I. The pathway also increases neuroinflammation and ALS neurodegenerative disease (Tan et al., 2022; Zanini et al., 2022). Furthermore, expansion of the repeat sequences of the *C9orf72* gene results in loss of function, which is associated with ALS, frontotemporal dementia, and autoimmune diseases. *C9orf72*-mutant cells are chromosomally unstable and fragile and are a source of endogenous damaged and immunostimulatory DNA which induces cGAS–STING pathway activation in ALS mice neurons, increasing neuroinflammatory damage(Mirceta et al., 2024).

Pharmacological inhibitors or agents against cGAS–STING-related genes can specifically reverse hyperactivation of the cGAS–STING pathway in microglia, which is induced by MSA-2 or ALS-associated toxic proteins, including TDP-43 and SOD1 (Duan et al., 2024; Zhan et al., 2025). Astrocytes of ALS possess neurotoxic phenotypes that are associated with a loss of their neurosupportive activities. There is evidence of reactive astrocytes in the spinal cord and cortex of patients with ALS, where these astrocytes produce inflammatory and pro-apoptotic mediators, thereby rendering motor neurons vulnerable to degeneration (Horiuchi et al., 2024). cGAS–STING signaling is emerging as an early pathological characteristic of astrocytes in ALS. Astrocyte overexpression of IFNs initiates the activation of the chemokine receptors of hippocampal neurons, disrupting presynaptic function and causing neuronal hyperexcitability, ultimately leading to cognitive impairments (Licht-Murava et al., 2023; Velasquez et al., 2024). Germeys et al. (2024) found that the Egln2 gene has the capacity to inhibit the activation of the cGAS–STING pathway in astrocytes, resulting in downregulation of ISG expression and protection of motor neurons, thereby inhibiting ALS disease progression. Qin et al. (2023) determined that the Listerin protein E3 ubiquitin ligase evokes TRIM27 recruitment to initiate K63-linked cGAS polyubiquitination. The process guarantees sorting and degradation of cGAS through an ESCRT-dependent pathway, alleviating neurobehavioral symptoms and pathological damage in mice with ALS.

In conclusion, various ALS genetic components such as TDP-43, SOD1, and C9ORF72 can induce cGAS–STING-evoked neuroinflammatory disease by releasing mtDNA and causing mitochondrial damage, ultimately leading to neurodegeneration.

#### Multiple sclerosis

Multiple sclerosis (MS) is a demyelinating autoimmune disease of the white matter of the CNS, accompanied by reactive gliosis, axonal injury, and neurodegeneration (Afzal et al., 2024). Invasion and recruitment of peripheral immune cells, including T cells, B cells, and macrophages, into the CNS lead to the release of numerous inflammatory cytokines and compromise the integrity of the blood-brain barrier (Orr and Steinman, 2025). This mechanism activates microglia and astrocytes, leading to neuroinflammation and MS onset (Kuhlmann et al., 2023). At end-stage diseases, resident immune cells in the CNS and B cells possibly derived from ectopic germinal centers facilitate disease progression and escalate neurodegenerative pathology (Klotz et al., 2023).

IFN-β has been established to be a successful therapeutic drug for MS and EAE, experimental autoimmune encephalomyelitis, and also a first-line clinical therapy for MS (Bellucci et al., 2023). IFN-β therapy controls the dialogue and interaction of immune cells, leading to reduced inflammatory responses, which in turn reduce the frequency of relapses and symptoms (Xavier et al., 2023). This therapy regulates the function and differentiation of immune cell subsets by directly altering the expression of surface receptors and survival conditions, and indirectly altering cytokine production. Notably, IFN-β significantly inhibits the Th1 polarization of CD4^+^ T cells by reducing myeloid and plasmacytoid dendritic cell-mediated pro-inflammatory cytokine production, while enhancing IL-10 secretion (O’Shea and Visconti, 2000). In addition, IFN-β inhibits differentiation of Th17 cells by inhibiting the production of pro-Th17 cytokines IL-1β and IL-23 through myeloid dendritic cells and B cells. It enhances the production of anti-Th17 cytokines IL-10 and IL-27 through myeloid and plasmacytoid dendritic cells, monocytes, and B cells, and therefore alleviates inflammation and autoimmunity (Ramgolam et al., 2011; Agasing et al., 2021; Karimi et al., 2021).

The cGAS–STING pathway plays specific regulatory roles across different cell types at various stages of MS pathogenesis. The expression of STING is highly elevated in neurons of the normal-appearing gray matter in progressive MS patients and active inflammatory lesions in relapsing MS patients (Woo et al., 2024). STING activation in microglia is increased in the acute phase of EAE but reduced in the chronic phase, suggesting that microglia exert distinct regulatory functions at different disease stages. In addition, in neurons under inflammatory conditions and glutamate-induced excitotoxicity, calcium-dependent STING activation can lead to autophagy-dependent ferroptosis through GPX4 degradation (Woo et al., 2024). By knocking out or inhibiting STING, the inflammatory cascade for neurodegenerative lesions can be relieved and hence reduce EAE severity and delay disease progression. Casella et al. also found that serine protease inhibitors induce IFN-β and IL-10 secretion by the activation of the cGAS–STING pathway in macrophages, with the net effect of suppressing autoimmune neuroinflammation in EAE (Casella et al., 2020). Mice lacking the volume-regulating anion channel LRRC8 have enhanced T cell-mediated immunity due to reduced uptake of cGAMP, reduced activation of STING, and reduced expression and signaling of the tumor suppressor p53. In EAE mice, there was increased infiltration of T cells and heightened autoimmune neuroinflammation in the central nervous system (Concepcion et al., 2022).

In summary, the function of the cGAS–STING pathway in MS/EAE is complex and is a debatable topic in relation to other neurodegenerative diseases. More research is warranted to further elucidate the role of cGAS–STING-related genes in MS /EAE pathogenesis.

### Movement disorder

#### Parkinson’s disease

Parkinson’s disease (PD) is a neurodegenerative disorder marked by the degeneration of dopaminergic neurons, progressive asymmetric bradykinesia, rigidity, resting tremor, and dystonia (Sul et al., 2024). The primary pathological features of PD include the death of dopaminergic neurons in the substantia nigra pars compacta and the formation of Lewy bodies, which are linked to the misfolding and pathological accumulation of α-synuclein (α-Syn) (Wiseman et al., 2024). A notable correlation has been identified between the upregulation of α-Syn and the STING protein in the substantia nigra pars compacta of PD patients (Hinkle et al., 2022). Key PD-related genes, such as leucine-rich repeat kinase 2 and those encoding PINK1 and/or Parkin, are crucial for the regulation of mitochondrial homeostasis (Nido et al., 2025). In macrophages lacking leucine-rich repeat kinase 2, mtDNA leakage into the cytosol is enhanced due to excessive mitochondrial fission mediated by dynamin-related protein 1, activating the cGAS–STING-IFN-I pathway and inducing neuroinflammation (Nido et al., 2025). Sliter et al. (2018) demonstrated that the knockout of Parkin and PINK1 exacerbates mitochondrial stress by impairing mitochondrial autophagy, leading to inflammation in PD mouse models through an mtDNA-cGAS–STING-dependent mechanism. Inhibiting STING can mitigate the loss of dopaminergic neurons and associated motor deficits. Additionally, metformin stabilizes mitochondrial function via Mfn2, reducing mtDNA release and leading to cGAS–STING inactivation, which effectively delays astrocyte senescence and improves dopaminergic neuronal damage and behavioral deficits in PD mouse models (Wang et al., 2024b).

It has been demonstrated that the accumulation of glucosylceramide in microglia not only induces leakage and activates the cGAS–STING pathway but also leads to lysosomal damage and impaired degradation of STING. This cascade of events further exacerbates STING-dependent neuroinflammation and neurodegeneration associated with the neuropathological progression of PD (Wang et al., 2024d). Mutations in VPS13C, which encodes a lipid transporter between the endoplasmic reticulum and lysosomes, have been linked to early-onset PD and result in lysosomal and mitochondrial dysfunction, subsequently triggering the cGAS–STING pathway (Hancock-Cerutti et al., 2022). Therefore, in addition to the activation of the mtDNA-cGAS–STING pathway, impaired lysosomal degradation of STING plays a significant role in the exacerbation of neuroinflammation in PD.

α-Syn is a central component in the pathogenesis of PD and is intricately associated with cGAS–STING-mediated neuroinflammation and neurodegeneration. Initially, the cGAS–STING pathway can promote α-Syn aggregation. Sustained activation of this pathway facilitates the assembly of the NLRP3 inflammasome, leading to neuroinflammation driven by the activation of microglia and astrocytes. This process ultimately results in the degeneration of dopaminergic neurons and further aggregation of α-Syn (Hinkle et al., 2022). This suggests that the interaction between STING and NLRP3 enhances α-Syn aggregation in PD. Simultaneously, α-Syn accumulation activates STING-dependent microglial activation. Moreover, inhibiting or knocking out STING reduces neuronal death in PD mouse models, alters the phenotypic transition of microglia and astrocytes from a homeostatic to an inflammatory state, delays cellular senescence and neurodegeneration, and has beneficial effects on motor dysfunction, reduces pathological α-Syn accumulation, and protects dopaminergic neurons (Zhao et al., 2021; Jiang et al., 2023a). Importantly, AIM2 negatively regulates microglial neuroinflammation by inhibiting AKT and IRF3 phosphorylation, thus restricting the activation of the cGAS–STING-TBK1 signaling pathway independently of the inflammasome (Rui et al., 2022). Conditional knockout of AIM2 in microglia exacerbates PD progression via the cGAS–STING pathway. Furthermore, Huang et al. demonstrated that hydrogen sulfide reduces cGAS–STING pathway activation in the hippocampus of PD rats, leading to decreased protein expression levels of cGAS and STING. This results in lower levels of tumor necrosis factor-α and IFN-β, thereby mitigating necroptosis and alleviating cognitive dysfunction associated with PD (Huang et al., 2025d).

Briefly, the cGAS–STING signaling pathway plays a potentially crucial role in PD pathogenesis through several interconnected mechanisms. These include facilitating α-Syn aggregation, inducing mitochondrial dysfunction, promoting neuroinflammation mediated by microglial and astrocyte activation, and ultimately contributing to neurodegeneration and neural injury.

#### Huntington’s disease

Huntington’s disease (HD) is an autosomal dominant neurodegenerative disorder characterized by motor disturbances, cognitive decline, and psychiatric symptoms (Xu et al., 2025a). The disorder arises from an abnormal expansion of the CAG trinucleotide repeat sequence within the huntingtin gene (HTT), leading to the production of mutant huntingtin (mHTT). This pathological polyglutamine (polyQ) protein exerts widespread cellular dysfunction, including transcriptional dysregulation, impaired DNA repair mechanisms, aberrant immune responses, and mitochondrial dysfunction (Bagherpoor Helabad et al., 2024).

Experiments have validated the cGAS–STING pathway as a key mediator of neuroinflammation and degenerative processes in HD. cGAS–STING-IRF3 signaling pathway activation has been consistently identified in both HD patients and model systems, where it correlates with pathological inflammation and synaptic dysfunction (Jauhari et al., 2020). Mechanistically, cells expressing mHTT have lower levels of interactions between mHTT and DNA mismatch repair factors, such as MLH1 and exonuclease 1. This interference leads to uninhibited exonuclease 1 activity, excessive DNA removal, and consequent DNA damage, further inducing cytosolic DNA accumulation. These events trigger cGAS–STING-dependent apoptosis pathways, a major mechanism of HD pathogenesis (Sun et al., 2024a). Furthermore, Toll-interacting protein, a STING stabilizer, plays a bimodal role in HD pathology. Toll-interacting protein encourages the clearance of polyQ aggregates; however, its binding with polyQ suppresses its association with STING, hence reducing the amount of STING protein and immune signaling (Pokatayev et al., 2020).

Therefore, the cGAS–STING pathway likely plays dual roles in HD. On one hand, cGAS–STING signaling contributes to the pathogenesis of HD. This is achieved through its association with mitochondrial stress, polyQ protein aggregation, and neuroinflammation. On the other hand, polyQ protein aggregation could induce sequestration of STING by Toll-interacting protein, which inactivates the cGAS–STING pathway, and leads to compromised immune signaling. Subsequent studies need to more clearly demonstrate the signalings through which the cGAS–STING pathway is involved in HD.

### Neuropathic pain

Neuropathic pain is a common chronic pain disorder caused by injury or disease of the somatosensory nervous system, significantly impacting quality of life (Perez-Sanchez et al., 2023). Trigeminal neuralgia, painful polyneuropathy, postherpetic neuralgia, and central post-stroke pain are a few examples (Alexandre et al., 2024). Most patients describe spontaneous or episodic pain, e.g., burning, tingling, or squeezing, that may be accompanied by evoked pain, particularly hypersensitivity to light touch and cold (Braffett et al., 2024). Under other circumstances, ectopic activity in areas such as neuromas at nerve endings, entrapped nerve or nerve root, spinal ganglia, and thalamus may form the foundation for spontaneous pain (Liu et al., 2023a; Baerwald et al., 2024). Evoked pain can spread to adjacent areas, with the underlying pathophysiological processes involving peripheral and central sensitization (Jiang et al., 2022). A series of intercellular communications and molecular signaling, such as changes in ion channels, activation of immune cells, glial cell-derived mediators, and epigenetic regulations, are accountable for these processes (Xie et al., 2022b; Xu et al., 2023; He et al., 2024).

Research has indicated M1 macrophages to be significant in the development of neuropathic pain (Guimarães et al., 2023). PNI is distinguished by the release of inflammatory mediators such as calcitonin gene-related peptide and substance P from nociceptive terminals which enhance vascular permeability, leading to local edema and ectopic discharges, ultimately triggering neuropathic pain (Wang et al., 2021a). Concurrently, damaged neurons release CCL2, enhancing Nav1.8 sodium channel activity in primary sensory neurons via a Gβγ-dependent mechanism (Belkouch et al., 2011). CCL2 also engages with macrophage receptors to trigger their recruitment to the site of injury (Xie et al., 2023a). Inflammatory mediators (IL-1β, tumor necrosis factor, and CXCL1) secreted by these macrophages can directly stimulate nociceptors to induce pain. These mediators also bind to corresponding receptors on non-nociceptor surfaces, generating receptor-mediated signal transduction via phosphorylation of MAPKs (p-p38 and p-ERK) and protein kinase A activation (Labuz et al., 2021; Huang et al., 2022). These kinases subsequently augment the activities of ion channels, such as transient receptor potential ankyrin subtype 1 protein/ transient receptor potential vanilloid receptor and voltage-gated sodium channels NaV1.7, NaV1.8, and NaV1.9, through posttranslational modifications. This creates hyperalgesia and increased excitability of nociceptors due to peripheral sensitization and ultimately leads to amplified sensitivity to pain (Anukrishna et al., 2023).

Notably, recent studies have demonstrated that the cGAS–STING-IFN-I signaling axis serves as a key regulatory pathway for neuropathic pain, modulating baseline pain sensitivity and chronic pain under various pathological conditions (Wu et al., 2022). Donnelly et al. (2021) found that STING-mediated IFN-I signaling in peripheral sensory neurons operates through autocrine and/or paracrine mechanisms. By binding to IFNAR, IFN-I inhibits the function of sodium and calcium channels, thereby suppressing nociceptor excitability. Mice deficient in STING- or IFN-I signaling are hypernociceptive and exhibit increased nociceptor excitability. Administration of STING agonists promoted the production of IFN-I and inhibited the excitability of nociceptors after nerve injury (Donnelly et al., 2021; Wang et al., 2021b). Zhang et al. (2023a) showed that STING activation in dorsal root ganglion neurons drove neuropathic pain through the TBK1/IKK/NF-κB/pro-inflammatory factor signaling pathway, which could be reversed by STING inhibitor. Thousand and one amino acids associated with neurodevelopmental disorders have recently been identified as potential upstream key molecules that regulate the activation of cGAS–STING pathway following chronic spinal cord injury (Zhang et al., 2023a).

In summary, the cGAS–STING pathway plays a complex and crucial role in neuropathic pain. The STING-IFN-I signaling pathway inhibits the excitability of nociceptors in sensory neurons by regulating ion channels. Meanwhile, the cGAS–STING pathway induces neuropathic pain by amplifying neuroinflammation through the activation of microglia.

### Neurological tumors

Neurological tumors are neoplasms that originate from neurons or glial cells, and they can be classified into two major categories: those of the central nervous system (such as gliomas and meningiomas) and those of the peripheral nervous system (such as schwannomas and neurofibromas) (Eichmüller et al., 2022). Due to their deep location and proximity to important neurovascular structures, neurological tumors are challenging to treat, often requiring a comprehensive intervention of surgery, radiotherapy, and chemotherapy (Annu et al., 2022). Currently, enhancing the anti-tumor immune response through the modulation of the cGAS–STING pathway has demonstrated promising therapeutic effects in the treatment of gliomas and neurofibromatosis (NF) (Berger et al., 2022; Somatilaka et al., 2024).

#### Glioma

Glioma, a primary intracranial malignant tumor arising from glial cells and neuronal precursor cells, exhibits a high incidence rate, frequent recurrence, and poor prognosis (Weller et al., 2024). The pathogenesis of glioma is closely linked to mutations in the histone H3.3-encoding gene (Leszczynska et al., 2024). These mutations disrupt DNA repair mechanisms, leading to the accumulation of extrachromosomal DNA. This accumulation activates the cGAS–STING pathway, triggering the release of immunostimulatory cytokines (Haase et al., 2022). The therapeutic potential of STING agonists is enhanced when combined with cell cycle checkpoint inhibitors. Notably, in gliomas, the STING gene frequently undergoes methylation-mediated silencing. This epigenetic modification significantly restricts the effectiveness of immune checkpoint inhibitors. By rectifying the dysfunction of STING signaling, it is expected to enhance anti-tumor immunity (Najem et al., 2024). Genetic ablation of the α isoform of the protein phosphatase-2A catalytic subunit in glioma cells amplifies the production of dsDNA and cGAS-IFN signaling, as well as upregulates major histocompatibility complex class I expression. This augmentation facilitates the infiltration of CD8^+^ T cells, natural killer cells, and dendritic cells, thereby bolstering antitumor immune responses (Mondal et al., 2023).

#### Neurofibromatosis

Neurofibromatosis is a group of genetic disorders characterized by the development of tumors along the nervous system, which can affect the brain, spinal cord, and peripheral nerves. The condition is predominantly classified into three distinct types: neurofibromatosis type 1 (NF1), neurofibromatosis type 2 (NF2), and schwannomatosis (Plotkin et al., 2022; Lin et al., 2024).

NF1 is caused by mutations in the *NF1*, which result in the downregulation of neurofibromin, leading to a spectrum of neurocutaneous disorders primarily characterized by neurofibromas (Lin et al., 2024). Somatilaka et al. (2024) demonstrated that the activation of STING in NF1 models enhanced the expression of CXCL10, thereby promoting the recruitment of T cells and dendritic cells. This process contributed to the proliferation and initiation of tumor cells. Malignant peripheral nerve sheath tumors are aggressive tumors and represent the leading cause of mortality in patients with NF1. In models of malignant peripheral nerve sheath tumors, combination therapy with STING agonists and immune checkpoint blockade facilitates the activation of effector T cells within the tumor microenvironment, thereby eliciting an antitumor immune response (Somatilaka et al., 2024). NF2 arises from heterozygous mutations in the *NF2* tumor suppressor gene and is clinically characterized by multiple tumor syndromes affecting the central or peripheral nervous system (Xu et al., 2024b). Recent investigations have revealed that patient-derived mutations within the FERM domain of *NF2* significantly suppress cGAS–STING signaling. This suppression leads to the inhibition of downstream IFN production, thereby impairing antitumor immune responses (Meng et al., 2021).

Notably, nanomedicines that combine the activation of the cGAS–STING pathway with the restoration of phosphatase and tensin homolog deleted on chromosome 10 have demonstrated promising immunotherapeutic effects, effectively mitigating the progression of brain metastasis (Zhao et al., 2025b). In summary, the activation or inhibition of STING exerts differential effects in specific stages of tumors. In neurological tumor models, it would be of significant interest to investigate the roles of cGAS–STING-related genes, as well as their impacts on tumor-associated cells.

## Regulation of Neural Regeneration through Tissue Engineering Techniques Targeting the cGAS–STING Pathway

Neurological injuries and disorders present significant therapeutic challenges, owing to their limited treatment efficacy, low cure rates, and frequent long-term sequelae, which collectively impose a substantial socioeconomic burden on affected individuals and healthcare systems. Recent advancements in tissue engineering, regenerative medicine, and precision medicine have catalyzed the development of novel therapeutic approaches aimed at mitigating neural damage and enhancing functional recovery (Liao et al., 2025). Among these innovations, biomaterials incorporating cGAS–STING-related genes have emerged as a promising strategy (Shi et al., 2022). In this section, we provide a comprehensive review of recent therapeutic advancements targeting the STING pathway, critically evaluate the limitations of current interventions, and propose actionable recommendations for optimizing future treatment paradigms.

### Current status of tissue engineering technologies for neurological disorders

Neurological diseases and injuries are often linked to high disability rates, severely undermining patients’ quality of life and imposing significant socioeconomic burdens on families and society. Despite research progress, effective therapeutic options remain scarce. Persistent neurological deficits frequently occur, seriously impeding patients’ well-being (Nicholson et al., 2020). Consequently, developing innovative therapeutic strategies that can accurately target neurodegeneration, neuroinflammation, and cellular senescence, while promoting neural morphological regeneration and functional restoration, has become a crucial interdisciplinary challenge. This endeavor spans the fields of materials science, neuroscience, tissue engineering, and regenerative medicine, representing a frontier of scientific and clinical importance (Henn et al., 2025).

**Figure 6:**
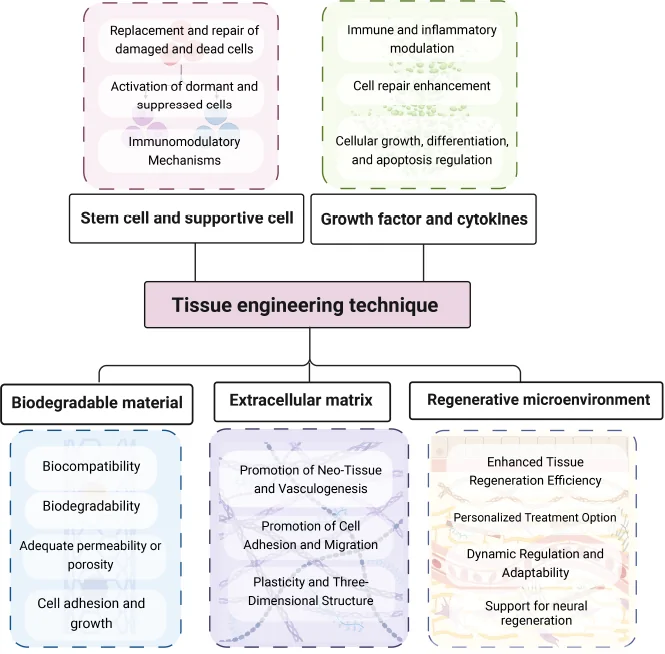
The five essential elements of crucial technologies in tissue engineering. The five essential elements of crucial technologies in tissue engineering are stem cell and supportive cell, growth factor and cytokine, biodegradable material, extracellular matrix, and regenerative microenvironment. Created with BioRender.com (https://BioRender.com/a03j256).

The development of innovative and effective therapeutic strategies for neurological diseases and injuries requires a multidisciplinary approach that integrates principles and techniques from biomaterials science and tissue engineering. Central to this approach are five critical elements of tissue engineering: biodegradable biomaterials, stem cells or supportive cells, growth factors or cytokines, extracellular matrix, and regenerative microenvironments (Gu, 2021; **Figure 6**). These components collectively provide the foundation for designing advanced therapies aimed at neural repair and functional restoration.

Biodegradable materials are defined as natural or synthetic biomedical substances capable of being degraded, absorbed, or excreted by the body through metabolic and biochemical processes when exposed to physiological fluids, acids, or enzymes (Kim et al., 2023). In recent years, a wide range of biodegradable materials has been employed as tissue engineering scaffolds to address peripheral nerve defects. These include synthetic polyesters such as polyglycolic acid, polylactic acid, poly (lactic-co-glycolic acid), polycaprolactone (PCL), and polyurethanes. Additionally, natural materials such as fibrin, collagen, keratin, alginate, chitin, chitosan, silk fibroin, extracellular matrix (ECM), and extracellular vesicles have been extensively utilized (Gu, 2021; Yu et al., 2024).

Stem cells are characterized by their unique capacity for unlimited or immortal self-renewal, as well as their ability to generate at least one type of highly specialized progeny cell. These cells exhibit remarkable self-renewal properties, allowing them to undergo continuous division and produce new stem cells, while also multipotent differentiation potential, enabling them to develop into diverse cell types (Mao et al., 2023). In tissue engineering, stem cell-based strategies are primarily focused on tissue repair and regeneration, cell culture and differentiation, and the development of scaffold materials (Zeng et al., 2025). Currently, different types of stem cells, such as adipose-derived stem cells, neural stem cells, and bone mesenchymal stem cells are predominantly centered in areas such as bone repair, muscle regeneration, and neural tissue repair (Zhou et al., 2023; Xu et al., 2024a; Zhu et al., 2024b).

Growth factors and cytokines are peptides or protein-based signaling molecules responsible for mediating intercellular communications and regulating significant cellular processes including proliferation, differentiation, migration, and gene expression. These molecules play essential physiological roles in maintaining tissue homeostasis and orchestrating biological responses (Cao et al., 2011). At the forefront of growth factors that play pivotal roles in neural regeneration are basic fibroblast growth factor, insulin-like growth factor, vascular endothelial growth factor, neurotrophin-3, nerve growth factor, and platelet-derived growth factor (Roam et al., 2015; Liao et al., 2025; Lu et al., 2025a). These bioactive molecules have been successfully applied in diverse tissue engineering applications designed to facilitate neural regeneration.

The ECM is a non-cellular, dynamic network present in all tissues and organs, synthesized and secreted by resident cells. It is primarily composed of collagen, non-collagenous proteins, elastin, proteoglycans, and glycosaminoglycans. In neural tissue, the ECM forms a membranous network that provides structural support, connectivity, and trophic support, while also facilitating tissue protection and repair. Additionally, it plays a critical regulatory role in modulating the functions of neurons, glial cells, immune cells, and endothelial cells (Hibbitts et al., 2022). In tissue engineering, the extracellular matrix serves dual functions: as a structural scaffold, it offers mechanical stability for cell anchorage and maintains organ morphology; and as a bioactive signaling platform, it cellular behavior and tissue development through interactions with cell surface receptors (Zhang et al., 2021).

An ideal regenerative microenvironment is most crucial for tissue engineering and regenerative medicine to be effective (Wang et al., 2022a). This microenvironment includes both the intrinsic microenvironment of the organ or tissue and the microenvironment modified by the introduction of implanted materials (Yang et al., 2023). The microenvironment for regenerative impacts cellular functions such as adhesion, migration, differentiation, proliferation, and communication on or inside the ECM or biomaterial interiors/surfaces (Gu et al., 2014). Bioactivity has been recognized in recent years as a significant property of biomaterials. Novel surface modification techniques, such as topographical modification, have greatly promoted the biocompatibility and activity of neural repair materials, thereby enabling the repair and regeneration of neural tissue (Zhu et al., 2021; Flamourakis et al., 2024). Moreover, Han et al. (2024a) assembled and fabricated a chiral hydrogel-based nerve conduit. In a rat sciatic nerve defect model, this hydrogel nerve conduit exhibited enhanced binding affinity for type IV collagen and demonstrated superior efficacy in promoting SC reprogramming, ultimately facilitating neural regeneration. Local regulation of the regenerative microenvironment of neural injury by the administration of bioactive materials can further improve conditions to favor neural regeneration (Yang et al., 2025c).

### Therapeutic strategies targeting cGAS–STING for modulating neurological disorders

**Figure 7:**
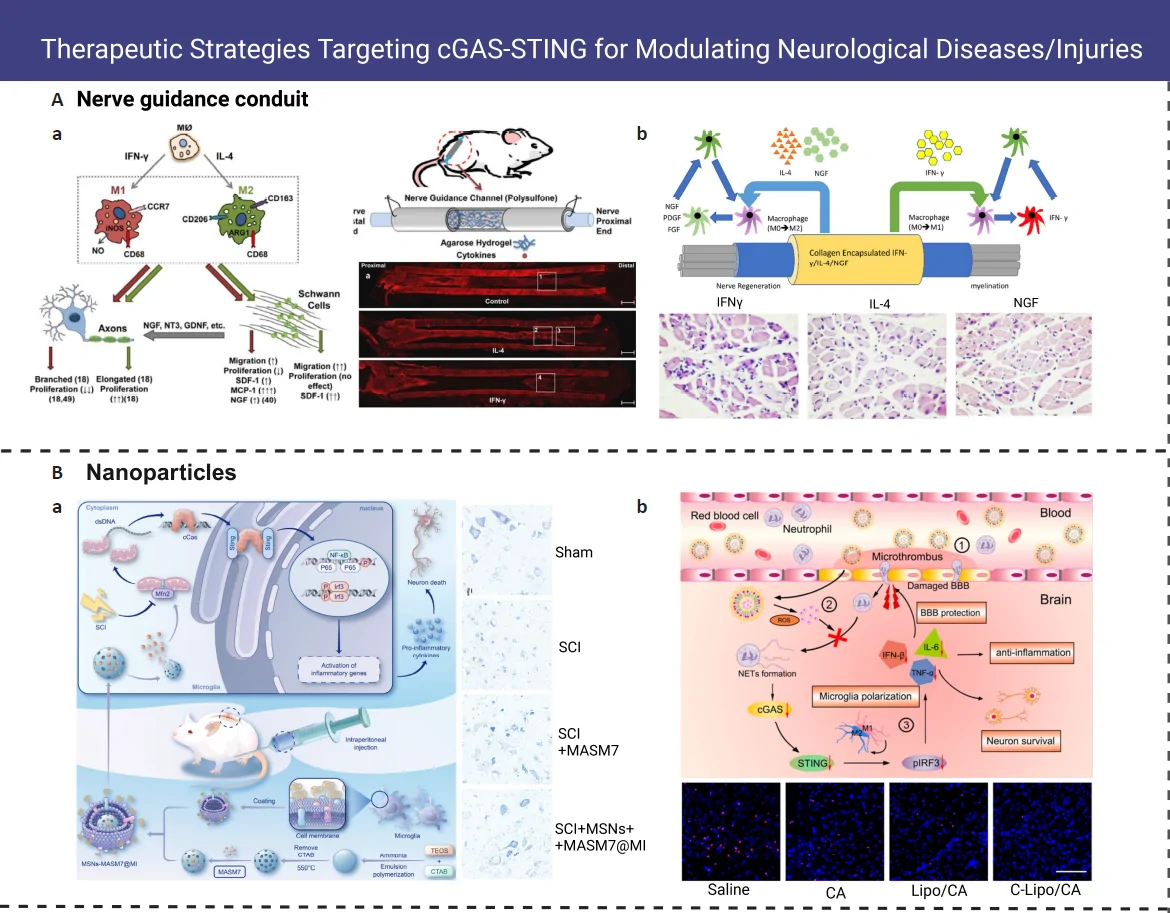
The application of therapeutic strategies targeting the cGAS–STING pathway in modulating neurological diseases/injuries. (A) Nerve guidance conduits: Neural catheters loaded with IFNγ stimulate the downstream activation of genes such as NF-κB and IRF3 through the activation of the cGAS–STING pathway in macrophages, thereby promoting the synthesis and release of inflammatory cytokines and polarizing macrophages towards M1 phenotype. a. Reproduced with permission from Mokarram et al. (2012) Copyright 2012, Elsevier. b. Reproduced with permission from Liao et al. (2019) Copyright 2019, Springer Nature. (B) Nanoparticles: Novel nanomaterials loaded with inhibitors of cGAS–STING-related genes suppress the inflammatory response in the region of neural injury, mitigating neural damage. a. Reproduced with permission from Wei et al. (2024) Copyright 2024, Wiley‐VCH GmbH. b. Reproduced with permission from Sun et al. (2023a) Copyright 2023, American Chemical Society. Created with BioRender.com (https://BioRender.com/f02k481).

To promote the regeneration of peripheral nerve defects more effectively, neural guidance conduits (NGCs) have been engineered in alignment with precision medicine principles. These conduits are capable of modulating key target genes, thereby facilitating functional peripheral nerve regeneration (Chen et al., 2021). Currently, novel tissue engineering scaffolds that regulate genes associated with the cGAS–STING pathway have been applied in the field of PNR. Researchers injected IFNγ into NGCs to bridge the sciatic nerve gap in rats, which activated a proinflammatory phenotype in macrophages, resulting in reduced SC recruitment and inferior neurological function recovery (Mokarram et al., 2012; Liao et al., 2019; **Figure 7A**). The activation of STING in neurons by IFNγ facilitates axon elongation, whereas IFNγ fosters the M1 polarization of macrophages, leading to local inflammatory responses. Chitosan, a biodegradable and biocompatible biomaterial commonly used to make NGCs (Deng et al., 2022; Jiang et al., 2023b; Zhao et al., 2023c; Zhou et al., 2023a), offers a promising platform. The activity of chitosan depends on various factors such as its degree of acetylation, molecular weight, size, polydispersity index, purity, and dosage (Reese et al., 2007; Lee et al., 2008; Da Silva et al., 2009; Bueter et al., 2011). Research has indicated that chitosan can stimulate the release of intracellular DNA. Biomaterials fabricated from chitosan can activate key proteins in the cGAS–STING pathway of macrophages, promoting the polarization of macrophages towards the M1 phenotype (Bueter et al., 2014; Carroll et al., 2016; Zhao et al., 2023d). In summary, the application of cGAS–STING-related gene modulators in PNR has been primarily focused on regulating immune responses and macrophage polarization. While foundational studies have demonstrated that STING activation promotes axonal extension, the effect has not yet been validated in the context of NGCs, largely due to the complexities associated with achieving precise neuronal modulation.

Biodegradable biomaterials engineered to modulate cGAS–STING-related genes have demonstrated significant potential in mitigating microglial-mediated inflammatory responses, reducing secondary inflammatory damage following SCI and stroke, and promoting subsequent neurological recovery. Wei et al. (2024) developed a microglial cell membrane-coated silica nanoparticle drug delivery system capable of delivering MASM7 to SCI sites. This system inhibits the activation of the Mfn2-mtDNA-cAS-STING axis, thereby alleviating inflammation and enhancing SCI recovery (**Figure 7B**). Similarly, Sun et al. (2023a) encapsulated the PAD4 inhibitor Cl-amidine within self-assembling liposome nanocarriers (C-Lipo/CA) modified with ROS-responsive polymers and fibrinogen-binding peptides for targeted delivery to ischemic lesions and stimulus-responsive drug release (**Figure 7B**). This system inhibits the neutrophil extracellular traps release process and further suppresses the cGAS–STING pathway in ischemic brain tissue, thereby reducing microglial-mediated inflammatory responses and promoting neuronal survival and subsequent neurorepair (Sun et al., 2023a). To combine STING inhibition with dsDNA clearance as an anti-inflammatory strategy, Zhu et al. (2023b) developed a novel nanomedicine that synergistically binds Ce enzymes mimicking DNase and STING inhibitors to alleviate immune inflammation in stroke and protect neurons.

In recent years, electrical stimulation has emerged as a highly promising physical therapeutic modality, demonstrating significant potential in disease treatment, wound healing, and mechanistic research due to its remarkable experimental outcomes (Mi et al., 2022). This approach can activate diverse intracellular signaling pathways, thereby modulating the intracellular microenvironment and subsequently influencing cellular processes such as migration, proliferation, and differentiation (Chiang et al., 2023). Huang et al. demonstrated that bilateral transcranial direct current stimulation effectively inhibits neuroinflammatory damage and reduces neuronal apoptosis by suppressing the cGAS–STING pathway, thereby enhancing cerebral injury recovery and improving motor function restoration (Huang et al., 2025b). When integrated with tissue engineering strategies, electrical stimulation has established itself as a tool in regenerative medicine (Boato et al., 2023). Furthermore, the development of biocompatible conductive materials has enabled the combination of electrical stimulation with tissue scaffolds incorporating cGAS–STING-related gene modulators (Guo et al., 2022; Kim et al., 2023). This synergistic approach fully leverages the complementary advantages of both techniques, positioning it as an optimal strategy in the field of neural regenerative medicine.

## Advances in Clinical Research

MS, as a representative neurological disorder, is an immune-mediated inflammatory demyelinating disease of the central nervous system, resulting in irreversible disability and affecting over 2 million individuals worldwide. The most prevalent form of MS is the relapsing-remitting subtype, accounting for 85%–90% of cases, which represents the primary target for most therapeutic interventions (Cohen et al., 2023). IFN-β, one of the drugs approved by the U.S. Food and Drug Administration (FDA) for the stable phase of relapsing-remitting MS, is available in five injectable formulations for its treatment (**Table 2**). While the clinical application of cGAS–STING-related genes in other neurological diseases or injuries remains limited, numerous drugs have demonstrated potential in preclinical studies. These include silibinin (Ping et al., 2024), metformin (Wang et al., 2024b), the neuroprotective agent Withaferin A (Zhao et al., 2021), and STING inhibitors (e.g., C-176 and H-151) (Sun et al., 2022a; Zhu et al., 2023b). These agents have been shown to reduce neuroinflammation and exert neuroprotective effects by inhibiting the cGAS–STING pathway. Further clinical validation of these findings is anticipated in future research. Furthermore, the therapeutic potential of additional STING modulators across diverse disease contexts will be systematically validated in forthcoming clinical investigations, thereby generating robust evidence to substantiate their efficacy.

**Table 2:** FDA-approved IFN medications for the treatment of MS

Drug name	Indication	Route and frequency of administration	Pivotal efficacy data	Common adverse events
IFNβ-1a (Avonex)	CIS+RMS	IM injection, once weekly	Relative reduction in 24-week CDP compared with placebo: 37%	Flu-like symptoms, muscle aches, asthenia, chills, and fever
PegIFNβ-1a (Plegidy)	CIS and RMS (1^st^ line)	SC injection, every 2 weeks	Relative reduction in ARR compared with placebo: 39%	Injection-site erythema, influenza-like illness, pyrexia, and headache
IFNβ-1b (Betaseron)	CIS and RMS (1^st^ line)	SC injection, every other day	Relative reduction in ARR compared withplacebo: 31%	Fever, chills, myalgia, sweating and malaise
IFNβ-1a (Rebif)	CIS and RMS (1^st^ line)	SC injection, 3 times weekly	Relative reduction in ARR compared with placebo: 33%	Injection-site inflammation, flu-like symptoms, rhinitis, and headache

ARR: Annualized replase rate; CDP: confirmed disability progression; CIS: clinically isolated syndrome; FDA: Food and Drug Administration; IFN: interferon; IM: intramuscular; MS: multiple sclerosis; RMS: relapsing forms of multiple sclerosis; SC: subcutaneous.

## New Techniques in Neural Regeneration Research

In recent years, the development of novel omics technologies has provided researchers with unprecedented insights into the dynamic changes of diverse cell populations during neural regeneration processes (Hao et al., 2022). Leveraging these advancements, the integration of core tissue engineering techniques with cutting-edge printing technologies, including three-dimensional (3D) and four-dimensional (4D) printing, has significantly enhanced the exploration of therapeutic effects and underlying mechanisms of various biomaterials in the treatment of neurological diseases and injuries (Sun et al., 2023b; Han et al., 2024b).

### Novel omics technologies

Single-cell sequencing has emerged as a powerful tool in neural regeneration research, providing unprecedented insights into the cellular heterogeneity and gene expression profiles within the nervous system (Nakamura et al., 2023). This technology enables the identification of different cell types and subtypes involved in neurological disorders and injuries, as well as key signatures of their transcriptional states (Matson et al., 2022). By analyzing the transcriptome of individual cells, researchers can uncover crucial molecular mechanisms underlying recovery from neurological diseases and neural regeneration after injury, such as the activation of regeneration-related signaling pathways, the regulation of gene expression, and interactions among different cell types (Xu et al., 2024c; Caporale et al., 2025).

Compared to single-cell RNA sequencing, spatial transcriptomics incorporates spatial location coordinates, enabling more comprehensive and multidimensional analysis of transcriptome data (Xu et al., 2024c). By integrating these two technologies, Wei et al. (2022) mapped the molecular cascades and potential cell lineages during regeneration and development in the telencephalon of axolotls following injury. Their findings highlighted the differentiation potential of ependymal glial cells as neural stem cells, which play a pivotal role in neural regeneration. They also proposed that brain injury regeneration partially recapitulates developmental processes. Li et al. (2022a) performed large-scale sequencing of mouse spinal cord tissue after SCI, revealing major pathological changes characterized by altered gene expressions. These changes included decreased expression of synaptic transmission-related genes, increased cell death, reactivation of the cell cycle, and activation of glial cell generation. Their study further identified microglia as critical players in SCI and long-term persistent injury, suggesting them as primary targets for future therapeutic drug development (Li et al., 2022a). Utilizing single-cell RNA sequencing, Sun et al. (2024c) identified a unique subset of bone marrow mesenchymal stem cells with enhanced axonal regeneration capabilities. They subsequently isolated exosomes derived from this subset and incorporated them into hydrogels to construct a sustained-release system, which improved neural functional recovery *in vivo*. Furthermore, other omics technologies, such as Bulk RNA sequencing, metabolomics, and proteomics (Wu et al., 2025), can be employed to gain deeper insights into the global expression changes of genes associated with neural regeneration, analyze the roles and regulatory pathways of metabolites in neural regeneration, and investigate the functions and interactions of proteins involved in the process.

In summary, the combined application of cellular omics and spatiotemporal omics enables the acquisition of high-resolution transcriptome data and the construction of comprehensive atlases of neural regeneration-related cells, providing a critical molecular foundation for identifying therapeutic targets for neurological diseases and injuries.

### Novel biomaterials

Tissue engineering scaffold materials serve as the material foundation for reconstructing tissues and organs through tissue engineering techniques and thus constitute one of the pivotal factors in tissue engineering research (Wei et al., 2023). With the rapid advancements in materials science and the increasing sophistication of tissue engineering techniques and theories, a variety of tissue engineering scaffolds have emerged. Currently, the biomaterials commonly used for fabricating tissue engineering scaffolds can be broadly classified into natural and synthetic categories. Natural materials primarily include chitosan, silk fibroin, and collagen, among others (Xue et al., 2023). Synthetic materials encompass silicone, PCL (Fang et al., 2024), and GelMA (Zhou et al., 2024). Tissue engineering scaffolds fabricated using natural materials to encapsulate gene activator inhibitors have demonstrated promising results in neural repair. Dong et al. (2022) developed an oriented nanofiber scaffold loaded with Deferoxamine (a hydrophilic angiogenic drug), up-regulating the expression of *HIF1-α* and *SDF-1α* genes and neural functional recovery. Tao et al. (2019) created a GelMA hydrogel conduit with MPEG-PCL nanoparticles encapsulating Hippo pathway inhibitors, which promoted recovery from PNI through continuous 3D printing. Wu et al. (2023) designed a GelMA/SF-MA hydrogel conduit incorporating high-loading 7,8-DHF prodrug nanoassemblies, enhancing neuronal regeneration and functional recovery. Yang et al. (2024b) introduced an adaptive hydrogel conduit (NC/DS@HPBGP) responsive to ROS and Ca^2+^, integrating gene modulators to support nerve regeneration and repair large peripheral nerve defects. It is anticipated that these materials will be integrated with STING-related gene regulators in the future, thereby facilitating efficient neural regeneration.

Due to the complexity of neural system structure and composition, the specificity of various neural tissue functions, and the dynamic regulatory requirements of the microenvironment for neural regeneration, single biomaterials often struggle to fully meet the needs of neural regeneration. Therefore, the preparation of multifunctional composite materials with specific functionalities, including but not limited to mitigating neuroinflammation and neurodegeneration, promoting neovascularization, facilitating glial cell migration, and axonal elongation, represents a major trend (Yao et al., 2024). Among the diverse functional scaffolds, metal-organic frameworks exhibit tremendous potential in neural regeneration engineering due to their high specific surface area, high porosity, excellent biocompatibility, and appropriate environmental sensitivity (Zhao et al., 2023b; Jiang et al., 2024). Researchers have developed a novel self-powered neural conduit by incorporating UIO-66 (a piezoelectric crystal material) and conductive reduced graphene oxide nanoparticles into PCL. This conduit improves oxidative stress and macrophage phenotypic switching in injured nerves, thereby enhancing oxidative metabolism and phenotypic modulation of infiltrated macrophages in the damaged neural microenvironment, ultimately promoting neural regeneration (Yao et al., 2023). Wang et al. (2023b) constructed a ZIF-8 nanoparticle delivery system loaded with microRNA-29a (miR-29a@ZIF-8) and incorporated it into a silk fibroin/gelatin-tyramine composite hydrogel conduit. This system promotes the transition of macrophages from the M1 phenotype to the M2 phenotype, significantly enhances myelination by Schwann cells, and promotes neuronal differentiation and axonal elongation of PC12 cells. Furthermore, Zhai et al. (2023) proposed a novel epidural microneedle patch composed of microneedle and β-cyclodextrin metal-organic frameworks. This patch can deliver methylprednisolone sodium succinate to the site of SCI in a controlled manner, effectively reducing post-SCI inflammation and improving neurological function. Chen et al. (2024a) designed a metal-organic framework nanozyme (MIL-101-NH2(Fe/Cu)) with ROS scavenging activity to encapsulate the neuroprotective agent rapamycin and modify its exterior with blood–brain barrier-targeting protein ligands. This design enhances drug retention and achieves controlled release within ischemic lesions, significantly reducing cerebral infarction volume and accelerating neurological recovery in a middle cerebral artery occlusion mouse model. Based on these studies, the outstanding performance of metal-organic scaffolds in inhibiting neuroinflammation, promoting myelination, and axonal elongation demonstrates their broad pre-clinical application potential in neural tissue engineering.

### Novel printing technologies

In recent years, 3D and 4D printing technologies have revolutionized the field of neural regeneration by enabling the precise fabrication of complex neural tissue scaffolds and conduits (Lin et al., 2023; Li et al., 2025b). 3D printing allows for the creation of scaffolds with customized porosity, surface topography, and mechanical properties that mimic the natural extracellular matrix, supporting neuronal cell adhesion, migration, and differentiation (Miao et al., 2020; Zheng et al., 2024). These scaffolds serve as ideal templates for neural regeneration, guiding the growth and organization of neural tissues (Yang et al., 2023; Kwon et al., 2024). Recently, numerous neural guidance conduits incorporating novel printing technologies have been designed and demonstrated to exhibit promising effects in promoting neural regeneration. Tao et al. (2019) created a methacrylated gelatin(GelMA) hydrogel conduit with MPEG-PCL nanoparticles encapsulating Hippo pathway inhibitors, which facilitated recovery from PNI through continuous 3D printing. Additionally, Chen et al. (2022a) designed a 3D-printed collagen/silk fibroin scaffold loaded with MSCs secretome that not only promotes the recovery of motor function but also enhances nerve fiber regeneration, myelin sheath regeneration, and accelerates the establishment of synaptic connections at the site of neural injury.

Furthermore, 4D printing expands the capabilities of 3D printing by introducing the concept of time-dependent shape changes. 4D printed scaffolds can undergo programmed transformations in response to external stimuli, such as changes in temperature or pH, allowing them to adapt to the dynamic environment of neural regeneration (Liu et al., 2024a). This adaptability enhances the integration of 4D-printed scaffolds with host tissues, fostering more effective neural regeneration. Utilizing the memory properties of 4D printing technology, Wang et al. (2024f) designed a novel artificial neural conduit that self-curls into a conduit structure upon body temperature triggering, enveloping the nerve stump and guiding cellular growth along microchannels, thereby facilitating the healing of peripheral nerve injuries. Additionally, Sun et al. (2023b) constructed a 4D orientation-dynamic scaffold based on shape-memory polymers, which precisely regulates the early and moderate proliferation of neurons spatially and temporally through the synergistic application of on-demand microtopography and deformation force-based mechanical stimulation, thereby promoting effective differentiation and axon formation.

In summary, the integration of single-cell sequencing, spatial transcriptomics, novel biomaterials, and 3D/4D printing technologies has significantly advanced neural regeneration research. These cutting-edge approaches have not only elucidated the molecular mechanisms and intercellular interactions critical to neural regeneration but also provided innovative strategies and methodologies for the design and fabrication of neural regeneration scaffolds.

## Existing Limitations and Prospects

The precise modulation of neuroimmune responses in neurological disorders and injuries has garnered substantial academic attention, with targeting the cGAS–STING pathway to finely regulate neural regeneration-associated cells emerging as a promising strategy to facilitate functional neural recovery. However, the precise roles of the cGAS–STING pathway in neurological disorders and injuries remain incompletely understood. The cGAS–STING pathway primarily influences neural regeneration by modulating neuroinflammation, neurodegeneration, cellular senescence, and cell death (Ding et al., 2022; Liu et al., 2025b). Activation of STING-related genes in microglia/macrophages and astrocytes induces the production of pro-inflammatory cytokines while impairing phagocytic function, resulting in a pro-inflammatory phenotype and clearance of pathogenic proteins (Zhu et al., 2023b). Nevertheless, the impact of STING-related gene activation on glial behavior and regenerative outcomes during nerve injuries remains unexplored. Moreover, although cGAS–STING pathway activation has been shown to elicit immune responses in neutrophils and T lymphocytes (Lu et al., 2022; Sun et al., 2023a), its exact role in neuroinflammation and neurodegeneration remains unclear. The involvement of ISGs induced by JAK-STAT pathway activation in the neurorestorative effects mediated by glial cells requires further investigation. Additionally, STING pathway activation is linked to multiple cell death mechanisms, exacerbating neuronal cell death (Victorelli et al., 2023). Activation of the cGAS–STING pathway in neurons has been demonstrated to the expression of regeneration-associated genes, upregulate tubulin, and facilitate growth cone formation and expansion, thereby supporting axonal elongation (Wang et al., 2023e). These findings highlight a facilitatory role of cGAS–STING-related genes in axon elongation, contrasting with previous emphasizing their detrimental effects on neural regeneration. This suggests that the role of cGAS–STING-related genes in promoting or inhibiting neural regeneration depends on the cell type and downstream pathways involved, revealing the complexity of the cGAS–STING pathway in neurological disorders and injuries. However, the specific pathways and genes involved in these processes require further elucidation to enable targeted modulation. Additionally, future research should investigate the role of the cGAS–STING pathway on angiogenesis in the nervous system.

To overcome the limitations inherent in current research, it is imperative to utilize advanced research methodologies, including spatial transcriptomics and single-cell sequencing, to elucidate the phenotypic transformations, roles, and mechanisms of the cGAS–STING pathway with precise temporal and spatial resolution in neurological diseases and injuries (Allen et al., 2023; Zhao et al., 2023a). Additionally, the exploration of mechanisms associated with the cGAS–STING pathway remains inadequate, necessitating further investigation into its roles in pertinent cells during neural regeneration, supported by comprehensive metabolomics and proteomics data. In the future, novel techniques from immunology and molecular biology can be integrated to provide a deeper and clearer understanding of the mechanisms underlying neurological diseases and injuries and the role played by the cGAS–STING pathway within this process (Morozzi et al., 2021; Wang et al., 2023e).

The integration of precision medicine represents an innovative approach to advancing neural tissue engineering in the future. Current research indicates that the cGAS–STING pathway plays a dual-phase role in neurological diseases and injuries. In the CNS, targeted inhibition of cGAS–STING pathway activation in glial cells has been shown to mitigate neuroinflammatory damage and promote functional neural recovery (Lv et al., 2024; Cui et al., 2025). Simultaneously, suppressing cGAS–STING pathway activation in neurons reduces programmed cell death and enhances neuronal survival (Wei et al., 2025). After PNI, activation of the cGAS–STING pathway in resident macrophages during the early stages of PNR may increase the population of M1 macrophages, accelerating the phagocytosis of myelin debris (Geng et al., 2023). Conversely, during the later stages of PNR, inhibition of this pathway promotes polarization toward the M2 phenotype, facilitating the release of anti-inflammatory factors and neurotrophic factors, thereby enhancing neural repair (Fan et al., 2025b). Based on the above theories, targeted regulation of the cGAS–STING pathway in regeneration-associated cells during neural regeneration represents a novel direction for the design of advanced biomaterials. However, there is limited research focused on developing therapeutic strategies that specifically target the cGAS–STING pathway to modulate neurological diseases and injuries. Moreover, existing biomaterials, when with agonists or antagonists of cGAS–STING-related genes, have not achieved fully satisfactory outcomes, as they fail to effectively promote optimal neuronal regeneration in the context of neurological diseases and injuries. This limitation may primarily stem from the lack of precise regulation of cGAS–STING signaling within specific cell types, as well as the absence of segmented modulation of STING-related gene activation and inhibition.

A variety of tissue engineering materials have been developed to target and regulate STING-related genes, thereby enhancing tissue regeneration. Various biomaterials, including novel nanoparticles (Zhu et al., 2023b; Luo et al., 2025), hydrogels (Chen et al., 2024b), ECM scaffolds (Pal et al., 2024), and electrospun nanofibers (Li et al., 2025a) have been developed to target and regulate STING-related genes, enhancing tissue regeneration. Nanoparticles loaded with a STING inhibitor alleviate microglial activation and reduce neuroinflammation (Shi et al., 2022; Zhu et al., 2023b). Pal et al. (2024) infused c-di-AMP into ECM scaffolds to promote a Th2-biased CD4^+^ T helper cell response and tissue healing. STING-related gene modulators have also been incorporated into hydrogel materials. Hydrogels loaded with siRNA (STING inhibitor) or c-di-AMP (STING activator) modulate T-cell infiltration and activation, thereby coordinating the immune response and promoting tissue regeneration (Chen et al., 2022b). Electrospun nanofiber materials targeting the JAK-STAT pathway have also shown promise in alleviating inflammation and accelerating wound healing (Xie et al., 2022a; Liu et al., 2023b). In the future, research should focus on mimicking the chemical composition of materials, growth factors, topological micro/nanostructures, and regenerative microenvironments, as well as elucidating the underlying rules and molecular regulatory mechanisms of the interactions between materials and organisms, and between tissue cells and materials. This represents the direction for the development of biomimetic tissue engineering technologies and clinically effective products.

**Figure 8:**
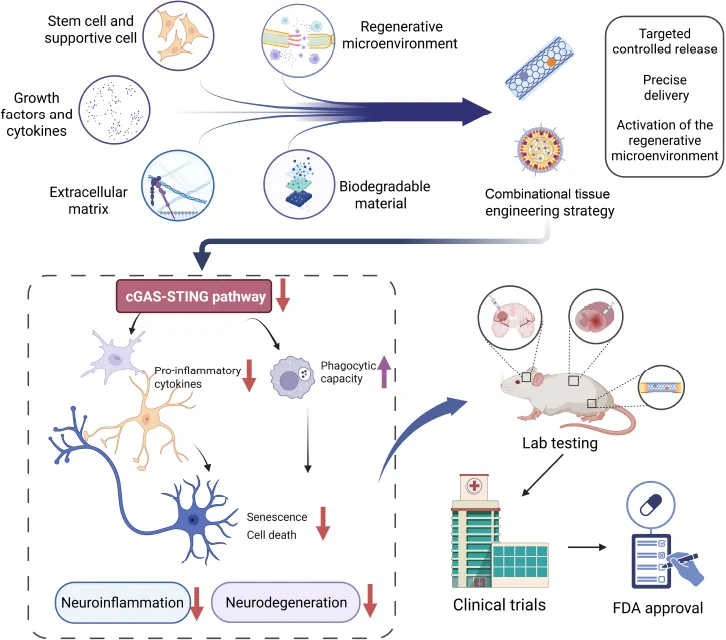
The development prospects of therapeutic strategies regulating the cGAS–STING pathway for neural regeneration. In the future, it will be necessary to integrate novel tissue engineering techniques to develop advanced tissue engineering strategies that incorporate targeted controlled release and precise delivery of inhibitors of cGAS–STING-related genes, with the ability to modulate the neural regeneration microenvironment. Leveraging advancements in precision medicine, these strategies aim to precisely regulate critical processes in neurological diseases/injuries, mitigating the inflammatory responses of microglia and astrocytes, and enhancing their clearance capabilities for cellular and myelin debris. This, in turn, aims to inhibit neuronal apoptosis and senescence, alleviate neuroinflammation and neurodegeneration, and ultimately promote neural regeneration. After validating the effectiveness of this therapeutic strategy in animal models, further clinical studies should be conducted to facilitate its eventual clinical translation. Created with BioRender.com (https://BioRender.com/isun8zb).

In the future, it is imperative to develop a system that integrates modulators and inhibitors targeting cGAS–STING-related genes with novel biodegradable scaffolds loaded with seed cells and neurotrophic factors, based on the concept of precision medicine and in conjunction with advanced targeted controlled-release technologies (**Figure 8**). This system holds promise for precise temporal and spatial regulation of the neural regeneration microenvironment, thereby potentially facilitating functional neural recovery. These formulations can be combined with emerging novel printing technologies, such as 3D and 4D printing (Sun et al., 2023b; Lin et al., 2025a), microwave printing (Aubert et al., 2024) and *in situ* printing (Xie et al., 2022b), to design neural regeneration scaffolds. 4D printing enables the responsive transformation of biomaterial structures in reaction to external stimuli such as temperature, humidity, light, and magnetic fields (Sun et al., 2023b). Incorporating STING-related gene modulators into printable materials involves selecting biocompatible materials for stability and bioactive substance release (Brannon et al., 2022), optimizing printing parameters (temperature, pressure, speed) to prevent loss of activators/inhibitors, and integrating controlled release systems to enhance biological effects (Afting et al., 2024). Additionally, 4D printing’s programmable deformation allows for precise, time- and location-controlled release of these modulators (Sun et al., 2023b), while adjusting printing parameters like layer thickness and speed can optimize cellular interaction and uptake (Xie et al., 2022c; Chen et al., 2025b). In the future, advanced therapeutic strategies utilizing the aforementioned technologies will enable precise, spatially-temporally controlled regulation of STING-related genes at various stages, fine-tuning cellular repair behavior to guide neural regeneration. Recently, artificial intelligence has shown great promise in biomaterials research by enabling efficient data analysis, reducing experimental costs and time, and driving innovation and personalization (Chen et al., 2023a). Notably, computer-aided design has been successfully translated into clinical practice, facilitating the creation of customized 3D models based on imaging of injured sites (Fay, 2020). The capabilities of AI for personalized, precise, and rapid model creation make it promising for therapeutic strategies in neural regeneration design.

## Conclusion

The cGAS–STING pathway plays a pivotal role in a wide range of neurological diseases and injuries. Overactivation of cGAS–STING-related genes in microglia/macrophages and glial cells exacerbates neuroinflammation and amplifies tissue damage. The deposition of neuropathological proteins triggers the activation of the cGAS–STING pathway within neural cells at the lesion site, resulting in increased release of neurotoxic transmitters, impaired clearance of pathological proteins, and an increase in senescent and dying cells, thereby accelerating the neurodegenerative process. In the future, elucidating the precise mechanisms underlying the regulation of neural regeneration by cGAS–STING-related genes is imperative for advancing strategies to promote neural regeneration based on precision medicine. Tissue engineering approaches that enable precise delivery and targeted controlled release of neural-regeneration-associated cells, along with spatiotemporal regulation of cGAS–STING-related genes within these cells, present a promising avenue for the development of therapeutic interventions tailored to the specific requirements of functional neural regeneration.

## Data Availability

*No additional data are available.*
